# Radical Reactions in Organic Synthesis: Exploring in-, on-, and with-Water Methods

**DOI:** 10.3390/molecules29030569

**Published:** 2024-01-23

**Authors:** Chryssostomos Chatgilialoglu, Sebastian Barata-Vallejo, Thanasis Gimisis

**Affiliations:** 1 Istituto per la Sintesi Organica e la Fotoreattività, Consiglio Nazionale delle Ricerche, 40129 Bologna, Italy; 2 Center of Advanced Technologies, Adam Mickiewicz University, 61-712 Poznan, Poland; 3 Facultad de Farmacia y Bioquímica, Departamento de Ciencias Químicas, Universidad de Buenos Aires, Junin 954, Buenos Aires CP 1113, Argentina; 4 Department of Chemistry, National and Kapodistrian University of Athens, 15771 Athens, Greece

**Keywords:** organic synthesis, radical reactions, water and aqueous media, on-water reactions, water coordination with Lewis acids, photocatalysis, bioinspired reactions

## Abstract

Radical reactions in water or aqueous media are important for organic synthesis, realizing high-yielding processes under non-toxic and environmentally friendly conditions. This overview includes (i) a general introduction to organic chemistry in water and aqueous media, (ii) synthetic approaches in, on, and with water as well as in heterogeneous phases, (iii) reactions of carbon-centered radicals with water (or deuterium oxide) activated through coordination with various Lewis acids, (iv) photocatalysis in water and aqueous media, and (v) synthetic applications bioinspired by naturally occurring processes. A wide range of chemical processes and synthetic strategies under different experimental conditions have been reviewed that lead to important functional group translocation and transformation reactions, leading to the preparation of complex molecules. These results reveal how water as a solvent/medium/reagent in radical chemistry has matured over the last two decades, with further discoveries anticipated in the near future.

## 1. Organic Reactions in Aqueous Media

The use of water as a solvent for organic reactions, initially proposed by Breslow [1], became very popular in the last three decades for various reasons, not only for being inexpensive and nontoxic but mainly for being an environmentally friendly substitute for organic solvents [2,3,4,5,6,7]. In addition, reactions “in water” may bring about many benefits, including easy product separation based on lower solubility in water, lack of toxicity and inflammability, high polarity, heat capacity, solubility of inorganic compounds, and, often, increased reaction rate and selectivity. A further boost to the use of this solvent came from the concept of reactivity “on water”, introduced by Sharpless and co-workers to describe reactions of water-insoluble organic compounds that take place in aqueous suspensions [8]. The development of organic microenvironments in the aqueous phase allows the reaction to proceed considerably faster in the presence of water than in organic solvents, and tremendous research progress on chemical reactions under aqueous conditions has been reported. Recently, the differences in reactivity between homogeneous and heterogeneous systems, that is, “in-water” and “on-water” systems, respectively, have attracted considerable attention, as testified by numerous reviews [8,9,10,11,12,13,14].

Despite these findings, the percentage of synthetic organic radical reactions carried out in the presence of water remained very small [15,16,17] until Macmillan and coworkers introduced the concept of photoredox catalysis [18]. In this review, we will present a variety of radical reactions where water exerts significant influence on the outcome.

## 2. Free Radicals in Water

### 2.1. Neutral Carbon-Centered Radicals Do Not React with Water

**Figure 1:**
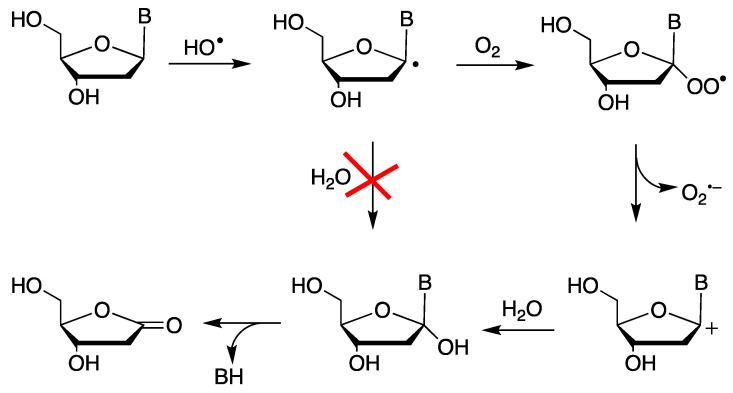
Mechanistic details of the formation of 2-deoxyribonolactone from 2′-deoxynucleosides: the C1′ radical is oxidized by addition to molecular oxygen, heterolytic cleavage with release of O_2_^•−^, followed by hydrolysis.

A large body of literature suggests that water does not react with neutral carbon-centered radicals. The products of their reactions in water are often the result of oxidation followed by a reaction with water. For example, exposure of DNA to enediyne antibiotics (e.g., NCS-Chrom) in the presence of oxygen leads to the formation of intermediate C1′ radicals and eventually to the creation of a 2-deoxyribonolactone lesion (Figure 1), as a result of C1′ carbon oxidation [19]. The detailed mechanism of the formation of 2-deoxyribonolactone, at the nucleoside level, revealed, through oxygen-18 labeling and a laser flash photolysis experiment, the involvement of heterolytic cleavage with the release of O_2_^•−^ (k = 1.5 × 10^4^ s^−1^), followed by hydrolysis [20,21].

**Figure 2:**
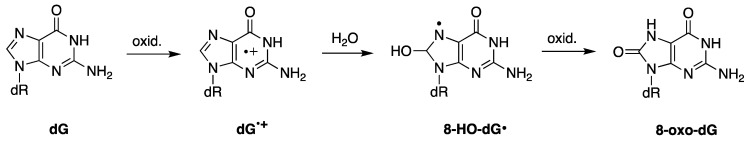
Oxidation of guanine moiety and the reaction with water, ultimately yielding 8-oxo-dG.

On the other hand, radical cations are prone to react with nucleophiles [22]. An example is the well-known guanine moiety G in nucleosides, ds-oligonucleotides, or DNA that can be oxidized to G^•+^ by a variety of oxidants, such as SO_4_^•−^, Br_2_^•−^, Cl_2_^•−^, and CO_3_^•−^, including various metal complexes. Figure 2 shows the case of 2′-deoxyguanosine (dG) and the resulting radical cation, which reacts with water to give the adduct 8-HO-dG^•^ in competition with a deprotonation step [23,24]. 8-HO-dG^•^ is a precursor of the 8-oxo-guanine moiety, the most relevant modification in genetic material [25]. 

Occasionally, it is reported that alkyl radicals may react with water directly by hydrogen abstraction [26]. Considering that the bond dissociation energy (BDE) of water is ca. 117 kcal mol^−1^, these reactions are highly endothermic and do not occur. However, it is well known that coordination with various Lewis acids can provide a substantial decrease in BDE and favor the reactions with an alkyl radical. We have summarized this area of research in Section 5 below.

### 2.2. Polar Effects and the Increase in Reaction Rates

The hydrogen abstraction from thiols by carbon-centered radicals (R^•^) is one of the most important reactions in free-radical chemistry. The intracellular concentration of glutathione (GSH) is in the range of 1–10 mM (depending on the cell type) and GSH serves as an H atom donor in the repair of R^•^ produced in biological systems [27]. 

Kinetic data on the reactions of thiols with R^•^ in organic, aqueous, and mixed solutions are numerous [28,29]. In organic solvents, for example, primary, secondary, or tertiary alkyl radicals abstract hydrogen atoms from Me(CH_2_)_7_SH with rate constants of ca. 10^7^ M^−1^ s^−1^, and an α-hydroxyl substituent has no net kinetic effect on the thiol trapping [30]. All the rate constants were found to be essentially the same for a specific thiol, despite the change in the reaction thermochemistry. On the other hand, a peculiarity of the radical substituents is found in aqueous solutions. Table 1 shows the rate constants for the reaction of the α-hydroxyalkyl radicals with 2-mercaptoethanol (Reaction (1)). The rate of reaction increases by replacing the H atom with the Me-group, despite the fact that the exothermicity of the reaction decreases in the same direction [29,31]. Other water-soluble alkanethiols behave similarly [32]. The observed order of reactivity, which is contrary to the BDE of the radical’s parents, has been discussed in terms of increasing polarization in the transition states [30,32].

HOCH_2_CH_2_SH + R^•^ → HOCH_2_CH_2_S^•^ + RH (1)

Free-radical-based chemistry in water has been thoroughly studied in radiation chemistry for several decades. Kinetic data for many radical reactions in water as the medium are obtained by pulse radiolysis [29]. It is also worth mentioning that through radical polymerization in water emulsions, millions of tons of polymer are prepared annually [33].

## 3. Synthetic Approaches Using Radical Intermediates in Water and Aqueous Media

**Figure 3:**
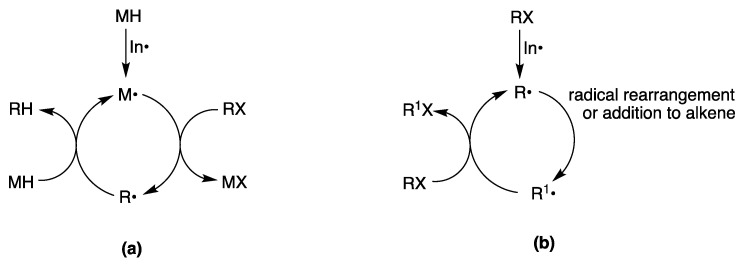
Radical chain mechanisms of (**a**) functional group reduction (X = atom or group) by a reducing agent MH; (**b**) halogen atom transfer in the new C–C bond formation.

Radical reactions have become popular among organic chemists with the introduction of reducing agents, like Bu_3_SnH or (TMS)_3_SiH, and led to a rapid growth in the number of organic transformations in organic solvents [34,35,36]. Figure 3a shows generic radical chain processes for the reduction of a functional group X by a reducing agent MH, where M^•^ radicals are generated by some initiation processes. The reduction chain is propagated from the removal of the X atom in the organic substrate (RX) by the M^•^ radical. The radical R^•^ then reacts with MH, giving the reduced product, RH, and a “fresh” M^•^ radical to propagate the chain. The chain is terminated by radical-radical combination or disproportionation reactions. The radical R^•^ may undergo further transformation, like intra- or intermolecular addition to an unsaturated moiety, giving a new radical, which is finally reduced by MH.

**Figure 4:**
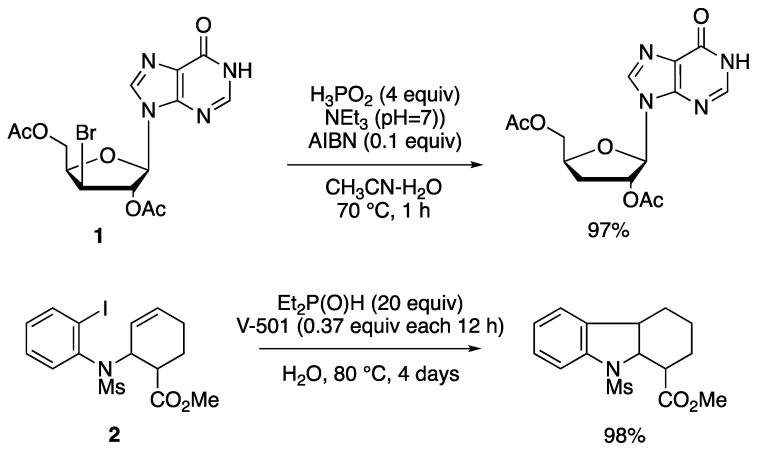
Radical-based reactions using H_3_PO_2_ or Et_2_P(O)H as reducing reagents in aqueous environments.

In earlier work, ad hoc synthesized water-soluble group 14 hydrides have been used for the reduction of halides [37,38], whereas Barton and coworkers introduced hypophosphorous acid (H_3_PO_2_) and its salts, or dialkyl phosphites, as radical-based reducing agents for organic halides and for the deoxygenation of alcohols through thionocarbonates or xanthates [39,40]. Hydrophobic substrates often limit the scope of these reactions and therefore the reactions employ a mixture of water and organic solvents or water and a phase-transfer agent [41]. The details of this research area are beyond the scope of this article. Several reviews have appeared in the last two decades and the reader is referred to these for further information [15,17,42]. Two reactions are reported in Figure 4 as examples connected to the mechanism mentioned above. The debromination of nucleoside **1** using H_3_PO_2_ in aqueous acetonitrile afforded the reduction product in 97% yield; since HBr is produced, triethylamine (Et_3_N) was used as a base to prevent the decomposition of the acid-labile substrate and product [43]. Using diethylphosphine oxide as the reducing reagent, the iodobenzene derivative **2**, in the presence of the water-soluble azo compound V-501 as the radical initiator, afforded the indole derivative in 98% yield, although large amounts of Et_2_P(O)H and V-501, and long reaction time, are needed [44].

**Figure 5:**
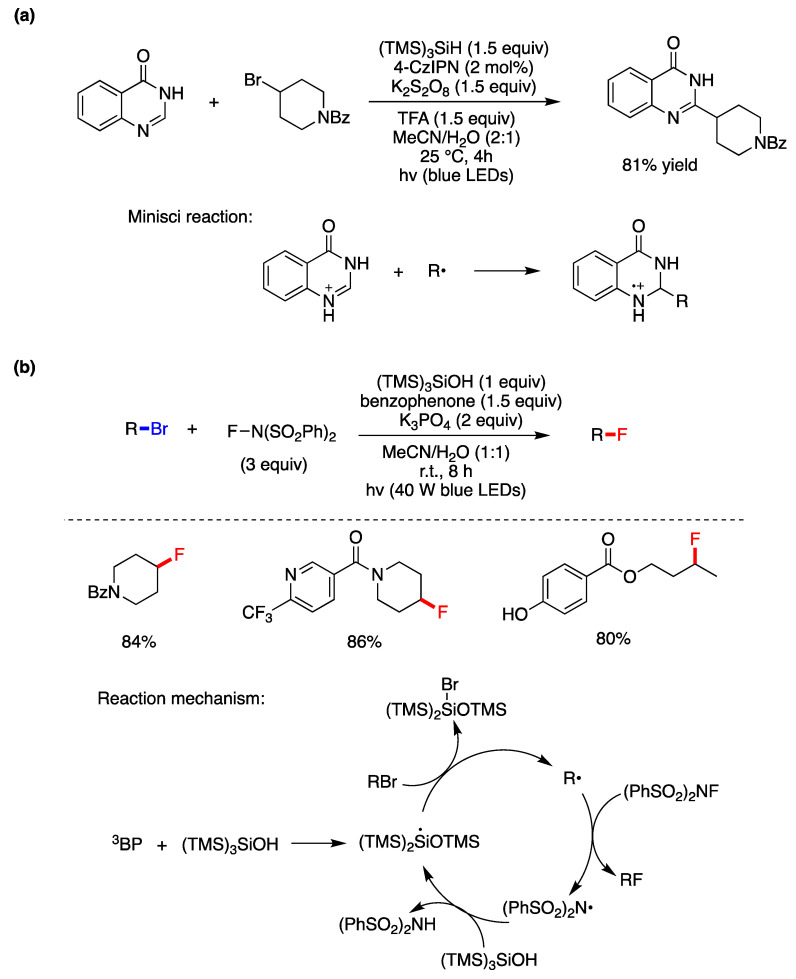
(**a**) Silyl-radical-mediated C–H alkylation of heterocycles with non-activated alkyl bromides. (**b**) Silyl-radical-mediated protocol for the fluorination of secondary alkyl bromides.

A protocol for the Minisci-type C-H alkylation of heteroarenes using the widely available and structurally diverse pool of non-activated alkyl bromides mediated by photoredox catalysis has been reported [45]. The example of quinazolinone and 4-bromopiperidine is shown in Figure 5a. It is proposed that the method utilizes the inexpensive oxidant K_2_S_2_O_8_ as a photocatalyst for the photoredox cycle and rearomatizes the *N*-heterocycle after the Minisci addition (inset of Figure 5a), whereas the R^•^ is generated by the reaction of (TMS)_3_Si^•^ with the alkyl bromide. The presence of water as a co-solvent considerably improved the yields by facilitating the dissolution of persulfate and purification of final products.

A synthetic strategy for the fluorination of alkyl bromides using *N*-fluorobenzenesulfonimide (NFSI) has been reported [46]. Figure 5b shows the optimized conditions and a few examples wherein good yields were obtained by radical chain propagation. The initiation step is the photoexcitation of benzophenone (BP) in its triplet state (^3^BP), which reacts with (TMS)_3_SiOH to generate the resulting silyl radical, via oxidation and deprotonation [47]. Subsequent bromine atom abstraction from RBr would then yield the corresponding alkyl radical. In its turn, fluorine atom transfer from NFSI leads to the corresponding nitrogen-centered radical which is polarity-matched to abstract a hydrogen atom from the hydridic silane (TMS)_3_SiOH, thereby propagating this chain mechanism via the silyl radical (TMS)_2_Si(^•^)OTMS generated by 1,2-SiMe_3_ shift from silicon to oxygen [46]. 

**Figure 6:**
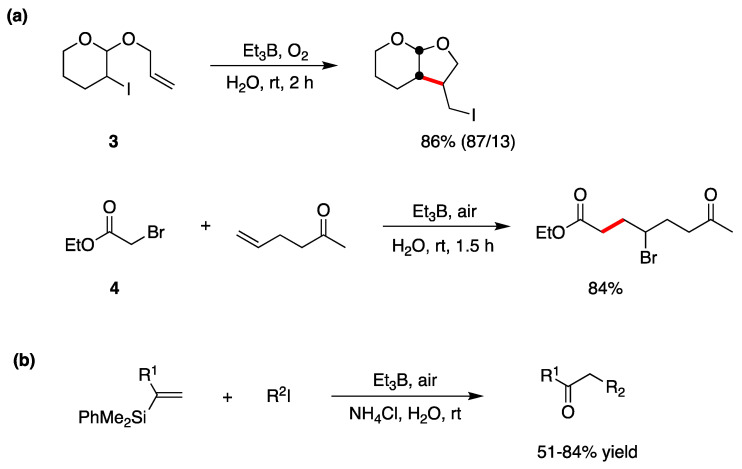
(**a**) Two examples of halogen atom transfer in intramolecular and intermolecular C–C bond formation, respectively. (**b**) Synthesis of ketones from alkenylsilanes under radical conditions with air as the oxidant.

Halogen atom transfer cyclization and addition have also been extensively studied in radical-based organic synthesis [48,49]. Triethylborane (Et_3_B) in the presence of a trace of O_2_ is an efficient radical initiator [50] and has been widely used for synthetic radical reactions. Et_3_B is also stable in water and aqueous media. Et_3_B-induced halogen atom transfer is used in a variety of radical reactions. Two examples are shown in Figure 6a, where the radical cyclization of the iodo derivative **3** and the intermolecular bromine atom transfer addition reaction of bromo ester **4** to an alkene proceed smoothly in good yields [15,51,52]. In the case of the iodo derivative **3**, by replacing H_2_O with MeOH/H_2_O (*v*/*v*, 3:1) as the solvent, a yield of 75% (88/12) was obtained. The reaction mechanism is reported in Figure 3b. Using similar approaches, imine [53] and alkenylsilane [54] derivatives are alkyl radical acceptors for the construction of new carbon–carbon bonds. In particular, a tandem intermolecular radical addition–oxidation sequence can convert vinylsilanes into ketones, via silyl hydroperoxides, in good yields (Figure 6b) [54].

**Figure 7:**
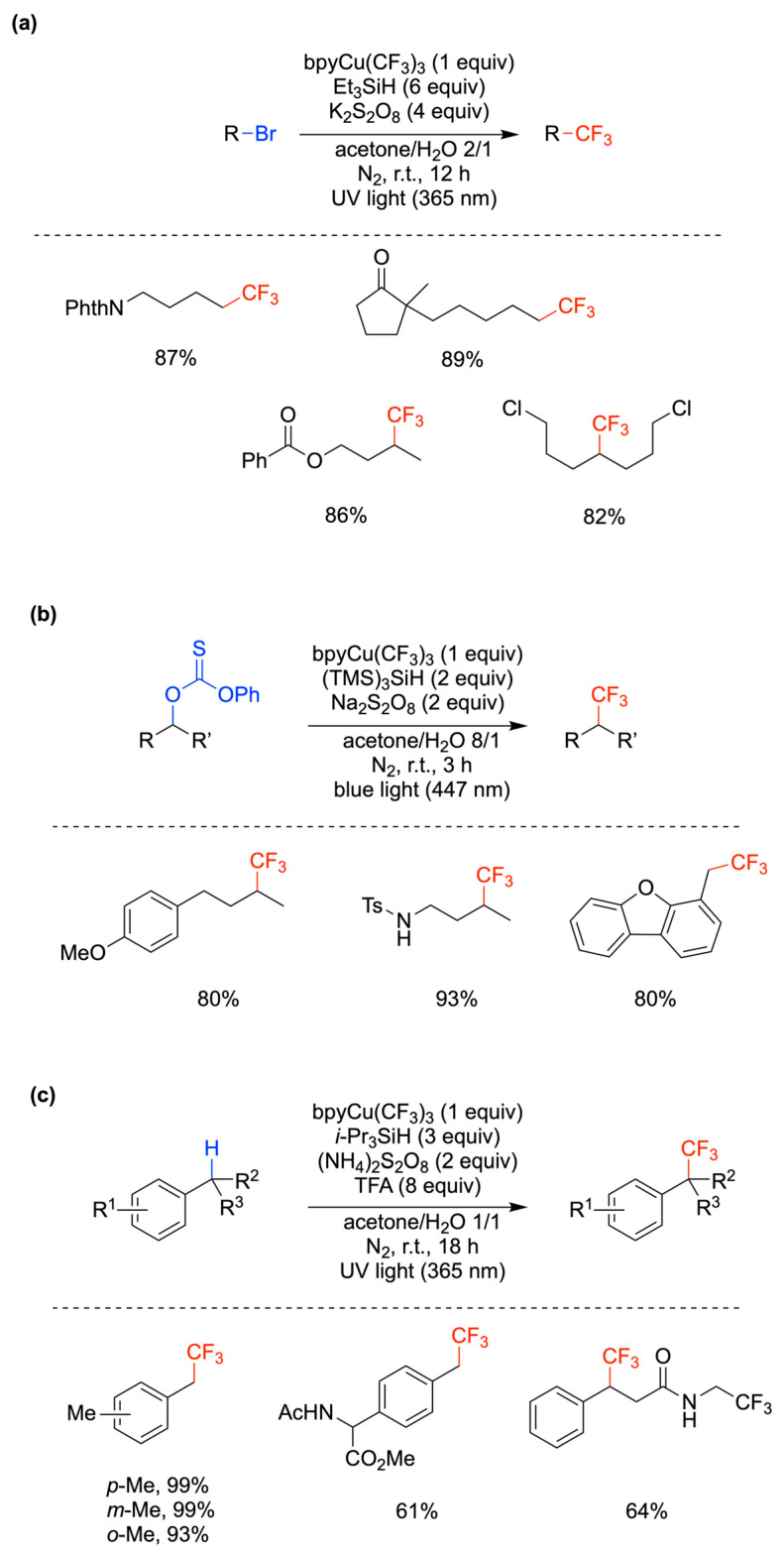
A light-driven, copper-mediated, site-selective trifluoromethylation for C(sp^3^)–CF_3_ formation in aqueous solution: (**a**) starting from alkyl bromides and Et_3_SiH; (**b**) starting from *O*-alkyl thiocarbonates and (TMS)_3_SiH; (**c**) starting from benzylic C–H and *i*-Pr_3_SiH; bpp = 2,2′-bipyridine.

The incorporation of CF_3_ groups into organic molecules has a profound effect on their lipophilicity, permeability, and metabolic stability, which are key elements in pharmaceuticals and agrochemicals [55]. Useful methods for the trifluoromethylation of a variety of organic compounds using (bpy)Cu^III^(CF_3_)_3_ (bpy = 2,2′-bipyridine) [56] in aqueous acetone have been recently reported and the yields varied from moderate to very good [57]. In Figure 7, three classes of reactions are reported, i.e., the trifluoromethylation of alkyl bromides [58], of alcohol via the *O*-alkyl thiocarbonates [59], and of benzylic C–H bonds [60], with the yields of some selected examples. Three different mechanistic schemes are proposed, although the key step involving trifluoromethyl group transfer from Cu^II^(CF_3_)_2_ intermediates to alkyl radicals is common to all. In the case of Figure 7a, it is suggested that the combination of Et_3_SiH/K_2_S_2_O_8_/light generates alkyl radicals through the reaction of Et_3_Si^•^ with RBr [58]. In the case of Figure 7b, the combination of (TMS)_3_SiH/Na_2_S_2_O_8_/light generates an alkyl radical through the reaction of (TMS)_3_Si^•^ with thiocarbonate, and, in parallel, the photolysis of (bpy)Cu^III^(CF_3_)_3_ produces CF_3_^•^ and Cu^II^(CF_3_)_2_ species; meanwhile, CF_3_^•^ generates (TMS)_3_Si^•^ by a second path, the Cu^II^(CF_3_)_2_ species react with the carbon-centered radical derived from the thiocarbonate derivative to yield the desired product [61]. In the case of Figure 7c, trifluoroacetic acid (TFA) was used as an additive to the combination of i-Pr_3_SiH/(NH_4_)_2_S_2_O_8_/light. It was suggested that UV light serves to generate SO_4_^•−^, which abstracts an H atom from the benzylic C–H bond, and for the homolysis of (bpy)Cu^III^(CF_3_)_3_ to form CF_3_^•^ and the active Cu^II^(CF_3_)_2_ species, whereas i-Pr_3_SiH serves for the quenching of CF_3_^•^, and perhaps the resulting silyl radical could form the benzylic radical [60]. In the above-described reactions, the medium acetone/H_2_O varied from 2/1 to 8/1 to 1/1, respectively, and the beneficial effect of water has been attributed to the improved solubility of K_2_S_2_O_8_ [58] or the Cu^II^ complex stabilization [60]. C(sp^2^)–H trifluoromethylation of aldehydes through acyl radicals under conditions similar to the ones for alkyl radicals (cf. Figure 7a) was also reported [61].

An analogous protocol has been reported for the decarboxylative trifluoromethylation of aliphatic carboxylic acids with (bpy)Cu^III^(CF_3_)_3_ using AgNO_3_ as the catalyst for the formation of alkyl radicals, and the combination ZnMe_2_/K_2_S_2_O_8_ in aqueous acetonitrile at 40 °C for 10 h [62].

## 4. Radical Reactions Occurring “on Water” and in a Heterogeneous Phase

Reactions “on water” involve water-insoluble organic compounds and take place in aqueous suspensions [8,9,10,11,12]. They have received considerable attention because of their high efficiency and application to straightforward synthetic protocols. In this section, we will give some examples of free radical chemistry useful to organic synthesis that proceeds in a heterogeneous phase. In particular, we will consider (i) “on-water” reactions with a lack of solubility of the reactants, and (ii) “in- and on-water” reactions with some reactants soluble in water and some others suspended, where an amphiphilic co-reactant is employed in order to transfer the radical reactivity.

**Figure 8:**
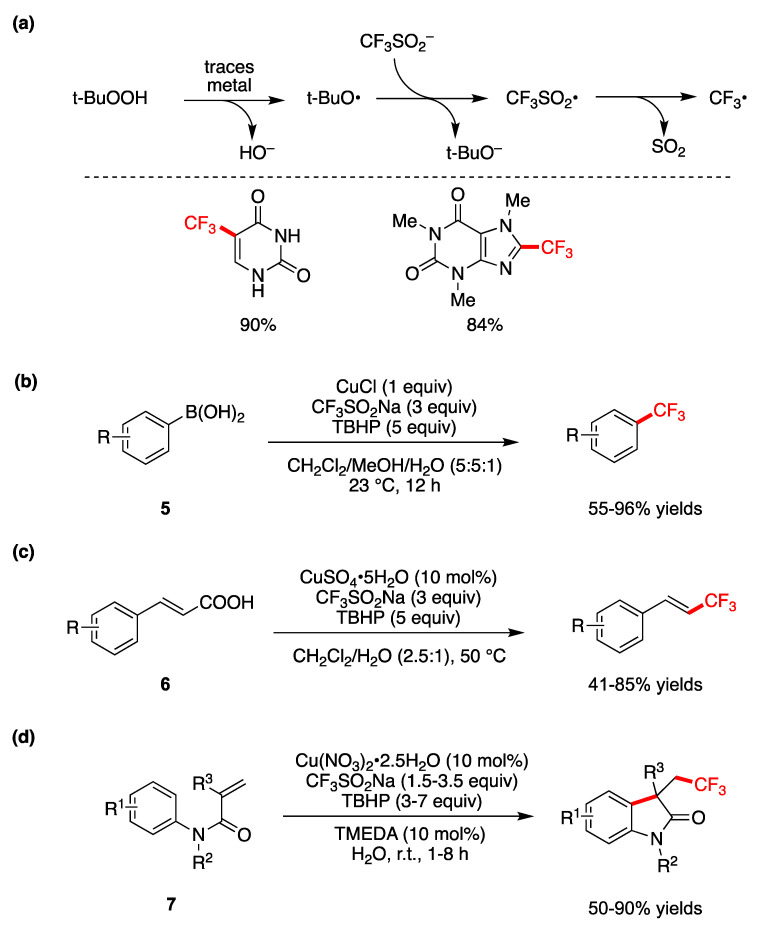
Copper-catalyzed trifluoromethylation using CF_3_SO_2_Na: (**a**) the mechanism of CF_3_^•^ generation from CF_3_SO_2_Na and tert-butyl hydroperoxide (TBHP), and the addition products of two heterocycles where the yields refer to a 1 g scale; (**b**) trifluoromethylation of aryl and heteroaryl boronic acids; (**c**) decarboxylative trifluoromethylation of cinnamic acids; (**d**) synthesis of a variety of CF_3_-containing oxindoles from *N*-arylacrylamides ‘‘on water’’ at room temperature.

Trifluoromethylation based on radical intermediates in water or aqueous medium has been developed by different groups; it functions broadly on a variety of substrates and demonstrates high functional group tolerance [63,64,65,66]. These reports are based on the discovery of a general procedure that combines CF_3_SO_2_Na (known as Langlois reagent), tert-butyl hydroperoxide (TBHP), and catalytic amounts of metal catalyst generating a CF_3_^•^ radical, as shown in Figure 8a [63]. Optimization of the method shows that catalysts like FeSO_4_ or CuSO_4_ in CH_2_Cl_2_/H_2_O (2.5:1) work well, but the trace metals found in the reagents were also sufficient. Similar results have been observed for the system with water alone as the solvent. Subsequent addition of the in situ-formed CF_3_^•^ to heterocycles or electron-rich arenes affords trifluoromethylation products. Two examples of trifluoromethylation of uracil and xanthine are shown in Figure 8a, obtained in CH_2_Cl_2_/H_2_O (2.5:1) and water, respectively, where the yields refer to a 1 g scale [63].

Trifluoromethylation of a variety of aryl and heteroaryl boronic acids **5** is shown in Figure 8b, using CuCl as the catalyst and CF_3_SO_2_Na and TBHP in aqueous medium at room temperature [64]. These reagents are suggested to react in situ to generate CF_3_^•^ as the active trifluoromethylating reagent. In the 18 examples, the corresponding products are obtained in moderate to high yields.

A CuSO_4_•5H_2_O-catalyzed decarboxylative trifluoromethylation of various α,β-unsaturated carboxylic acids **6** by using CF_3_SO_2_Na and TBHP was developed in aqueous medium at 50 °C (Figure 8c) [65]. In particular, the decarboxylative coupling reactions of various cinnamic acids start with the addition of a CF_3_^•^ radical at the α-position of the double bond, then proceed via the elimination of carbon dioxide and Cu(I) to generate the product. In 24 examples, the corresponding products are obtained in moderate to high yields and an *E*/*Z* ratio of up to 99:1. The same authors also show a similar radical process for iron-catalyzed difluoromethylation of arylsubstituted acrylic acids by using (CF_2_HSO_2_)_2_Zn [65].

A Cu-catalyzed sequential difunctionalization/trifluoromethylation of *N*-arylacrylamides **7** that leads to oxindole derivatives has been reported “on water” at room temperature (Figure 8d) [66]. Optimized conditions were found for Cu(NO_3_)_2_•2.5H_2_O and TMEDA used in a 1:1 ratio (TMEDA = Tetramethylethylenediammine) as the catalyst. Yields of 18 examples vary from moderate to high, depending on the substituents R^1^, R^2^, and R^3^, the quantities of CF_3_SO_2_Na and TBHP, and the reaction time [66]. This protocol for the introduction of the CF_3_-group exhibits several noteworthy features, such as an inexpensive and readily available catalyst and trifluoromethylating reagent, and in some cases, reaction conditions that rely on water and ambient temperatures, in air.

**Figure 9:**
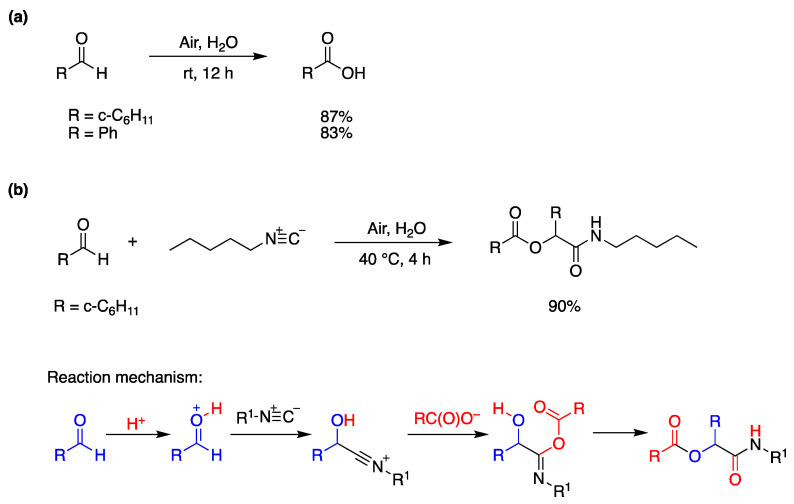
(**a**) Aldehyde oxidation “on water”. (**b**) Tandem aldehyde oxidation/Passerini reaction “on water” including the reaction mechanism.

Hydrophobic, aliphatic, and aromatic aldehydes undergo facile oxidation upon simply stirring their aqueous emulsions in air to obtain the corresponding carboxylic acids in high yields. Two examples are given in Figure 9a, where the water-insoluble products can be extracted with CH_2_Cl_2_ and isolated after solvent evaporation [67]. The mechanism in Figure 9a involves the formation of acyl peroxide via a radical chain (addition of acyl radical to molecular oxygen followed by hydrogen abstraction from aldehyde) and subsequent reaction with aldehyde to afford the carboxylic acid. The authors further explored the possibility of using an aldehyde as the source of both carbonyl and ester functions in the Passerini reaction, shown in Figure 9b. In this example, the hydrophobic cyclohexyl aldehyde and pentyl isocyanide in a 3:1 ratio, for 4 h, at 40 °C, afforded the desired product via the mechanism shown in Figure 9b [67].

**Figure 10:**
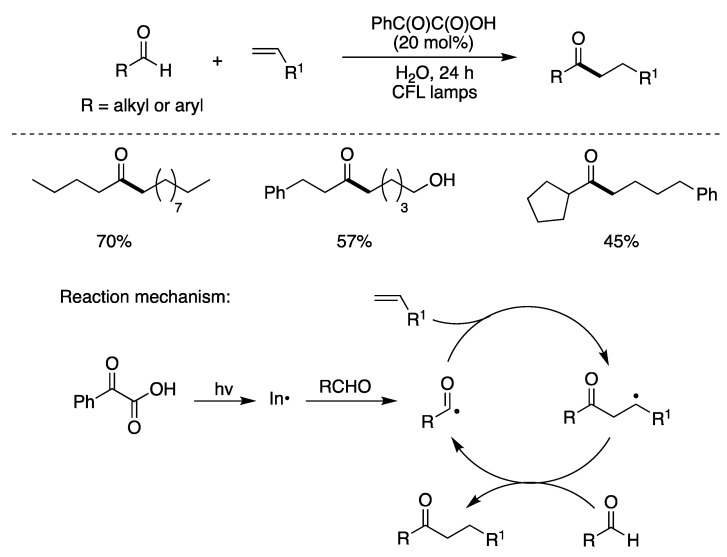
Photochemical hydroacylation of unactivated olefins “on water”.

A mild, metal-free photochemical method for the hydroacylation of olefins has also been developed [26,68]. This protocol was employed for a variety of aliphatic and aromatic aldehydes and olefins. Figure 10 shows the optimized conditions and a few examples obtained in moderate to good yields, by radical chain propagation. The initiation step is the photoexcitation of phenylglyoxylic acid that generates PhC(O)^•^ and exchanges aldehydic H with RC(=O)H [26]. The propagation step is straightforward, involving the addition of acyl radical to alkene and the adduct alkyl radical abstracting the aldehydic hydrogen to complete the chain reaction [69].

**Figure 11:**
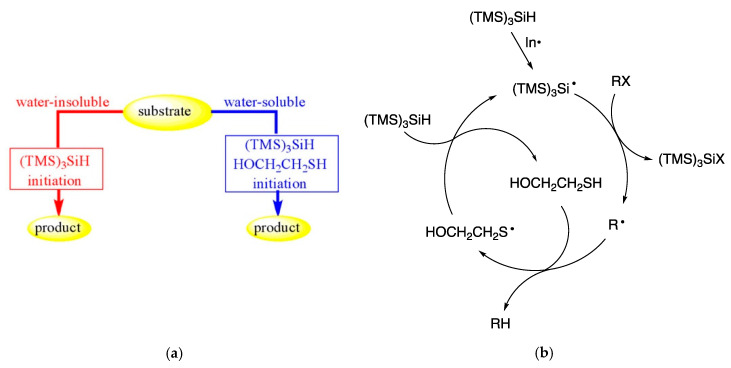
The use of (TMS)_3_SiH as a reducing agent in an aqueous environment: (**a**) two different experimental approaches depending on whether the substrate is water-insoluble or water-soluble, where an amphiphilic thiol is needed; (**b**) mechanism of the reduction of a functional group (X = atom or group) by the (TMS)_3_SiH/HOCH_2_CH_2_SH coupling, where the hydrogen atom donor to R^•^ is the thiol.

Tris(trimethylsilyl)silane, (TMS)_3_SiH, is a well-known reducing agent in organic chemistry based on free radical reactivity in organic solvents [36,70,71,72]. It is worth noting that (TMS)_3_SiH is insoluble and does not decompose in water, even under reflux for hours, at 100 °C [73,74]. Two methods have been reported for the use of (TMS)_3_SiH as a reducing agent in an aqueous environment depending on the hydrophilic or hydrophobic character of substrates (Figure 11a). For water-soluble substrates, the amphiphilic β-mercaptoethanol was necessary as a co-reactant. As the initiator, the water-insoluble 1,1′-azobis(cyclohexane-carbonitrile) (ACCN; half-life of 2.33 h at 100 °C) was found to give the best performance with both hydrophobic and hydrophilic substrates [73,74].

The reduction of organohalides and different thiocarbonyl alcohol derivatives (Barton–McCombie reaction) was obtained successfully. When water-insoluble materials such as substrate, reagents, and initiator are suspended in an aqueous medium and vigorously stirred, an efficient vortex and dispersion can be created, allowing for interaction between the materials. The reduction chain proceeds in the usual way: (i) removal of the X atom or group from the organic substrate (RX) by the (TMS)_3_Si^•^ radical, and (ii) the reaction of the radical R^•^ with the silane giving the reduced product, RH, and a “fresh” (TMS)_3_Si^•^ radical to propagate the chain. In the hydrosilylation of water-insoluble alkenes, alkynes, and aldehydes suspended together with (TMS)_3_SiH and the radical initiator ACCN, in an aqueous medium at 100 °C under vigorous stirring, these substrates can be transformed into (TMS)_3_Si-containing compounds in good yields [75].

**Figure 12:**
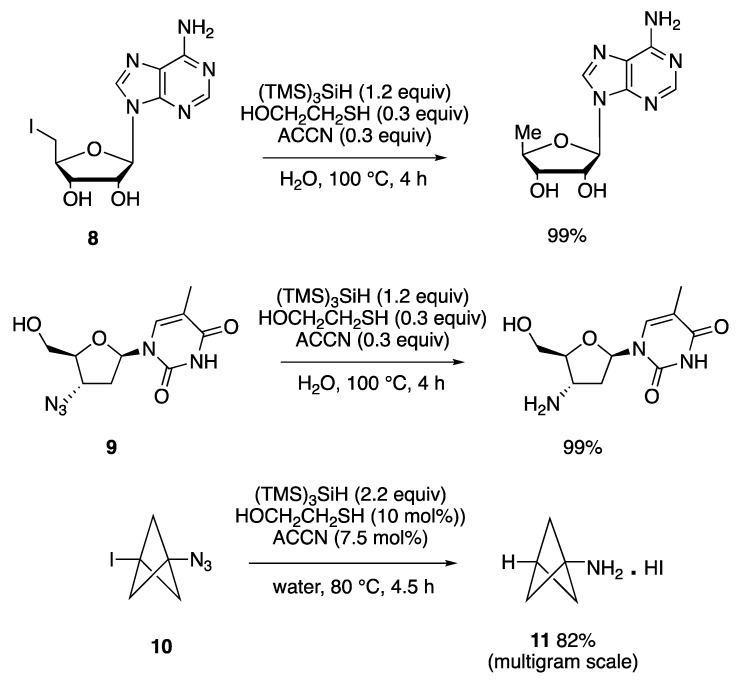
Radical reduction of a variety of compounds by the (TMS)_3_SiH/HOCH_2_CH_2_SH coupling.

In Figure 12, three examples of high-yield reductions, in the presence of (TMS)_3_SiH (TTMSS) and 2-mercaptoethanol, at reflux, with ACCN initiation, are shown. For example, the reduction of water-soluble iodo-containing compound **8** involved the amphiphilic thiol acting as the hydrogen donor, which is then regenerated by a reaction of the resulting thiyl radical with silane (Figure 11b) [74]. A water-soluble alkyl and aryl azides can be reduced to the corresponding primary amines by the same reagent coupling, in aqueous media and in the presence of a lipophilic initiator, with nucleoside derivative **9** being an example. The mechanistic steps for this transformation include the addition of a silyl radical to the azide function, followed by the liberation of nitrogen and the formation of a silyl-substituted aminyl radical, which abstracts a hydrogen atom from the thiol. The hydrolysis of the silylamine occurs rapidly in water and affords the amine as the final product [74].

The azido reactivity is utilized for the synthesis of bicyclo[1.1.1]-pentan-1-amine (**11**), a unique and important fragment in medicinal chemistry. By the aforementioned procedures, this scaffold was produced from precursor **10**, which contains both iodo and azide functions. TTMSS (2 equiv) is required for fully reducing both moieties. Under optimized conditions, the practicality of the protocol was consistent on multigram scales, in good yields (78–82%) [76].

**Figure 13:**
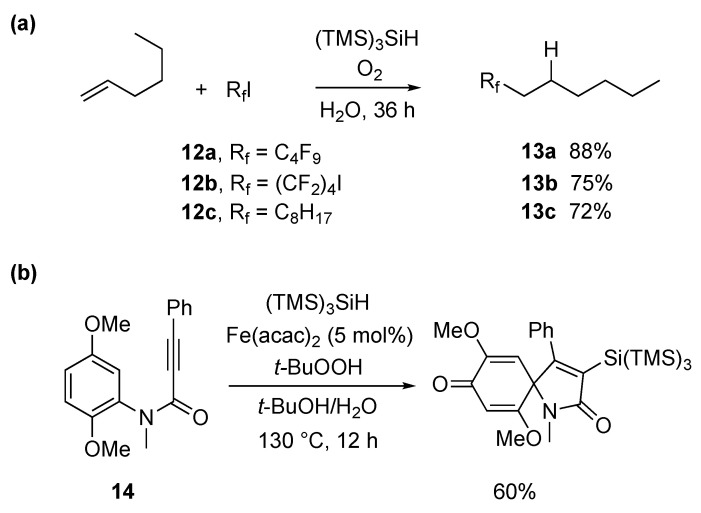
Carbon–carbon bond formation mediated by (TMS)_3_SiH: (**a**) perfluoroalkylation of olefins; (**b**) synthesis of a nitrogen-containing heterocycle.

The coupling of polyfluoroalkyl radicals to olefins was found to be mediated by the (TMS)_3_Si^•^ radical on water and was also successfully employed for the construction of C-C bonds in the synthesis of perfluoroalkylated aliphatic compounds (Figure 13a) [77]. Initiation of the process as in the above examples formed the silyl radical ready to abstract the iodine atom from R_f_I precursors (**12**), thus generating the corresponding perfluoroalkyl radicals. The addition of the latter to 1-hexene, followed by fast reduction of the resulting C-centered radical by TTMSS, led to the desired perfluoroalkylated products **13** in good yields. Fast addition of the electrophilic radical R_f_^•^ to olefins, as compared to its reduction by TTMSS, is crucial for the effectiveness of this process.

In Figure 13b, the formation of a nitrogen-containing heterocycle, through a radical cascade reaction, in good yield, is shown [78]. The reaction proceeds through the formation of a t-BuO^•^ radical, by a Fe(II)-catalyzed decomposition of t-BuOOH, followed by the abstraction of a hydrogen atom from TTMSS. The addition of (TMS)_3_Si^•^ to alkynone **14**, followed by a 5-exo-trig cyclization at the ipso position, leads to a cyclohexadienyl radical, which is finally trapped by a Fe(III)–OH species, to regenerate the Fe(II) catalyst and afford the final product.

**Figure 14:**
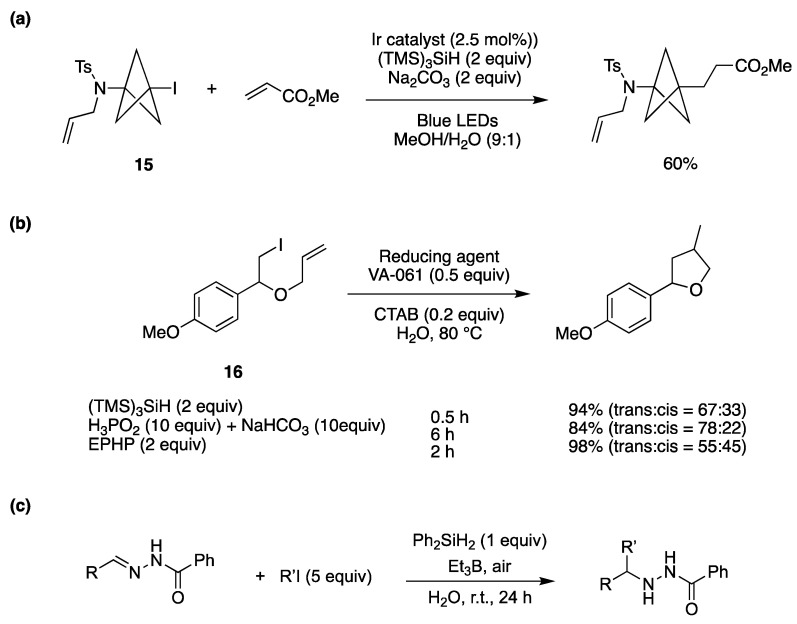
(**a**) An example of the synthesis of N,C-difunctionalized bicyclo[1.1.1]pentanes. (**b**) Comparison of three protocols using (TMS)_3_SiH, the water-soluble 1-ethylpiperidine hypophosphite (EPHP), or H_3_PO_2_/NaHCO_3_, respectively. (**c**) Radical addition to the C=N bond in hydrazone derivatives.

The (TMS)_3_Si^•^ radical can be generated by direct oxidation of the corresponding silane ([*E*^ox^ (TMS)_3_SiH/(TMS)_3_SiH^•+^ = +0.73 V vs. SCE in MeCN) followed by deprotonation [79]. A visible light-promoted hydrosilylation of alkynes by (TMS)_3_SiH has been achieved by using catalytic amounts of Eosin Y (1 mol %) as a photocatalyst, the thiol i-Pr_3_SiSH as a radical quencher, and potassium carbonate as a base additive in dioxane/H_2_O (100/1 *v*/*v*) [79]. The corresponding alkenylsilanes were provided with high regio- and stereoselectivity in the reactions of various terminal and internal alkynes. The role of H_2_O is to facilitate the regeneration of thiol by protonation of thiolate derived from the reaction of thiyl radical with reduced Eosin Y. The iodo derivative **15** has been successfully functionalized via a (TMS)_3_SiH-mediated carbon–carbon bond formation [80]. An example of the optimization procedure is reported in Figure 14a, using the iridium catalyst Ir[(dF(CF_3_)ppy)_2_(dtbbpy)]PF_6_ as the radical initiator. The reaction is thought to proceed via a common radical chain reaction. Interestingly, using MeOH as the solvent, the yield is quite low, but the presence of 10% H_2_O afforded the highest yield.

Figure 14b shows the hydrophobic substrate **16** in water with a combination of water-soluble radical initiator 2,2′-azobis[2-(2-imidazolin-2-yl)propane] (VA-061) and surfactant cetyltrimethylammonium bromide (CTAB). For comparison, three protocols are reported using the hydrophobic (TMS)_3_SiH, the water-soluble 1-ethylpiperidine hypophosphite (EPHP), and water-soluble H_3_PO_2_/NaHCO_3_, as the chain carrier. With (TMS)_3_SiH, the target product was obtained in good yield within a short period of time [41].

The radical addition/reduction reaction of alkyl iodides (R’I) to the C=N bond of hydrazones has been developed “on water” (Figure 14c) [81]. The reaction was optimized for R = Ph and R’ = *i*-Pr and was faster “on water” than in organic solvents, reaching 92% yield. Good yields were obtained for a variety of substituents on both reagents. The developed protocol can be applied to the synthesis of 3-substituted isoindolinone derivatives. Extension of this protocol to radical addition to the C=N bond in hydrazone derivatives of α-ketoesters has also been described [82].

It is also worth mentioning the achievement of a radical polymerization reaction involving Lewis pair catalysts “on water”, at room temperature [83]. Various poly(meth)-acrylates, diethyl acrylamide, and 4-vinylpyridine, initiated by Lewis pair catalysts, specifically PPh_3_/Cu(OTf)_2_ or PPh_3_/Sn(OTf)_2_, are prepared with significantly higher efficiency than the same reactions in organic solvents or in bulk.

## 5. Reaction of Carbon-Centered Radicals with Water-Activated by Lewis Acids

The most accurate value for the bond dissociation energy (BDE) of the H–OH bond homolytic cleavage has been estimated as 117.59 kcal/mol [84]. It has been estimated that even the highly reactive phenyl radical reaction with water is endothermic [85]. These facts preclude the reaction of water with carbon-free radicals under standard free radical reaction conditions and have allowed for all the chemistry in and on water described in the previous sections. Starting around 2005, there have been increasing reports of water (or deuterium oxide) activation through coordination with various Lewis acids that decrease the H–OH BDE of water and make the participation of water in radical chain reactions possible.

The subject of coordination-induced bond weakening of X–H bonds (where X is mainly O, N, or C) is one of increasing importance in recent years [86]. Below, we describe each system, with emphasis on those that have been well studied in terms of mechanism and applications in synthesis. We are focusing on the application of H–OH homolytic bond-weakening methods and their application in synthesis.

Although proton-coupled electron transfer (PCET) reactions are only formal hydrogen atom transfer (HAT) reactions and do not involve a homolytic H–OH bond cleavage, reactions of this type are also included, as they constitute formal HAT processes. In a recent review of PCET reagent thermochemistry by R.G. Agarwal et al. [87], there is an interesting and enlightening discussion on the concept of coordination-induced bond weakening and how it correlates with the reducing ability of the coordinating metal. The related work of Cuerva [88] on titanium (III) and the works of J. M. Mayer [89] and R. A. Flowers [90] on samarium (II) will be described below. But first, the chemistry of boron and the works by the groups of J. L. Wood [91] and P. Renaud [92] will be described, which initiated the studies in this field and eventually in the field of thiol-assisted radical reductions [93]. This section will be completed with the most recent reports on germanium [94], molybdenum [95], and bismuth [96], which have not been applied yet in synthesis, as well as a very recent report on phosphorus [97].

### 5.1. Boron Derivatives

**Figure 15:**
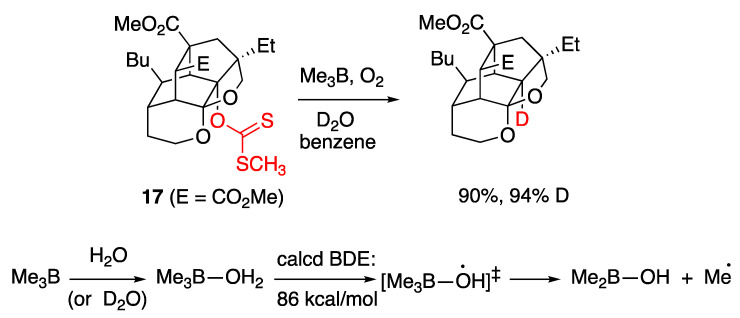
Thioxanthate radical deoxygenation by J. L. Wood and coworkers, and proposed water “activation” [91].

The first system which claimed H-OH bond weakening through coordination, was initially described in 2005 by Wood and coworkers [91]. It involved a variant of the Barton–McCombie deoxygenation of xanthates in the presence of trialkylboranes, utilizing water or deuterium oxide as the hydrogen or deuterium atom source, respectively. Τreatment of a xanthate **17** with trimethylborane and an excess of D_2_O, under free radical producing conditions, generated the deoxygenated product in high yield (90%) and high deuterium incorporation (94%) (Figure 15). The authors [91] proposed that a free radical mechanism was operating and that D (or H) atom abstraction from D_2_O (or H_2_O) was assisted by complexation with trimethylborane, a proposal corroborated by ab initio calculations that estimated an 86 kcal/mol BDE for the H–OH bond when oxygen was bound to boron. The reaction was also shown to operate in the presence of other alkyl boranes and other xanthate substrates [91] and was later expanded to the reduction of alkyl iodides with H_2_O (but not D_2_O) [98].

**Figure 16:**
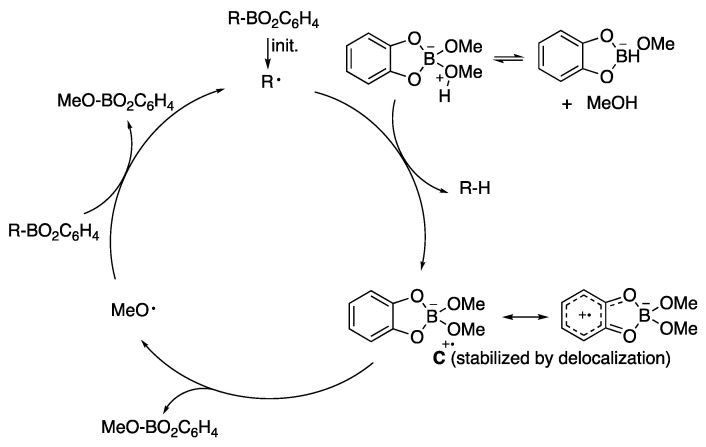
Mechanistic proposal for the MeO–H bond weakening [99].

Around the same time, P. Renaud and coworkers [99] made a similar observation in the radical reduction of β-alkylcatecholboranes to alkanes proposing a MeO–H bond weakening when coordinated with a catecholborate intermediate. The mechanistic scheme proposed (Figure 16) was corroborated by deuteration experiments. Also, J. Boivin and coworkers reported, in a series of papers [100,101,102], that the reduction of *S*-alkylthionocarbonates, including 2-oxoalkyl derivatives, could be effected under radical conditions in the presence of triethylborane and air. This was combined with mechanistic studies that suggested that the origin of the hydrogen atom could be an OH group complexed with boron, as suggested by Wood, the solvent, or triethylborane since the reduction gave good yields even under anhydrous conditions. J. Jin and M. Newcomb [103] came to a similar conclusion and in addition, they measured the apparent rate of the reaction with either water or methanol using a radical clock and determined that the reaction is entropically disfavored and its rate is enhanced at low temperatures. The low entropy of the reaction was later corroborated and measured (Δ*S*° ~ −24 kcal/mol) by P. Renaud and coworkers [104], who also corrected the rate constants when they determined that (a) the complex Et_3_B·MeOH is formed in very small quantities (1–2%) and (b) increasing complexation with excess methanol (or water) addition to species such as Et_3_B·MeO-H⋯O(H)Me leads to a diminished reduced hydrogen donor character that explains all the radical chemistry that has been observed in or on water in the presence of Et_3_B.

**Figure 17:**
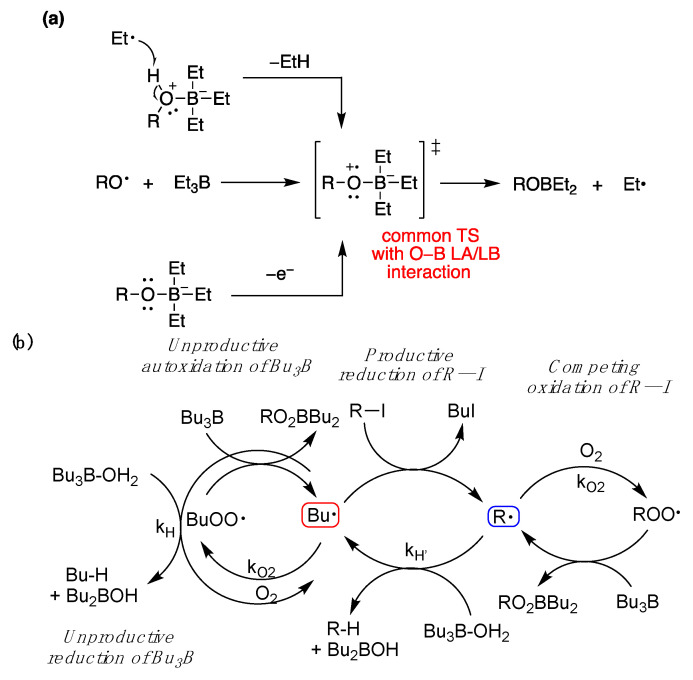
(**a**) Valence bond representation of common-features TSs accessed from three different classes of reactions. Though these are radical reactions, the TS features a Lewis acid/base interaction between O and B. (**b**) Three chains interlock with the reduction deiodination chain and with each other [50].

In 2016, Dennis P. Curran and T. R. McFadden [50] helped to explain initiation with Et_3_B and O_2_ by defining two regimes, one of low and one of high oxygen concentration. They explained that the radical reduction of alkyl iodides with Et_3_B·OH_2_ [98] and the chemistry described above by P. Renaud and J. Boivin worked through a common-features transition state (Figure 17a) primed for ejection of an alkyl radical in a rather inefficient radical chain, operating under the high-oxygen regime. They also predicted that reductions with Et_3_B·RO–H, (R = H or Me), where a Lewis acid/Lewis base interaction between oxygen and boron is important, would not be able to be optimized due to three other destructive radical chains interlocked with the reduction deiodination chain (Figure 17b), unless the radicals Bu^•^ and R^•^ exhibited different selectivities.

**Figure 18:**
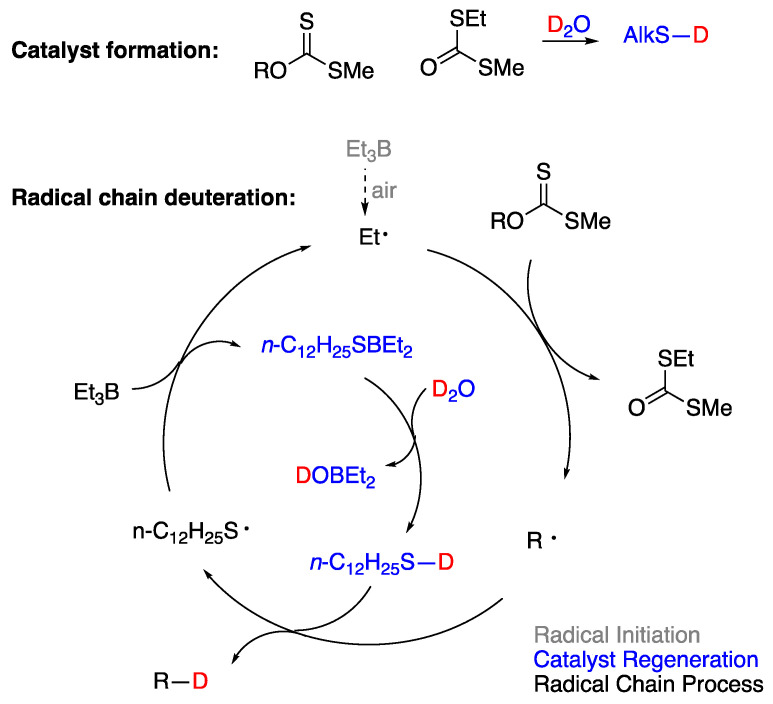
Revised mechanism for the deoxygenation of xanthate employing D_2_O as the deuterium atom source [92].

In 2018, thirteen years after the initial report by J. L. Wood [91], P. Renaud and coworkers [92] revisited the reaction of xanthates and alkyl iodides with Et_3_B·OH_2_ or Et_3_B·OD_2_. They corroborated Curran’s proposal that hydrogen (or deuterium) atom abstraction from H_2_O (or D_2_O) was only a minor and inefficient radical chain path [50]. They discovered that the main reduction involved, in the case of xanthates, in situ adventitious generation of thiol from partial hydrolysis of xanthates. The thiol acted as a catalyst for the majority of the observed reaction products, according to Figure 18. In this mechanistic scheme, the role of D_2_O was to simply generate a deuterated thiol through a proton–deuterium exchange that would provide the deuterium atom required in an otherwise typical radical reduction mechanism. Alkyl iodides could also be efficiently deuterated in the presence of thiols and D_2_O [92]. In the light of P. Renaud’s work, every mechanistic proposal of radical deuteration with D_2_O in an environment where there is a possibility of a generation of a thiyl radical or related intermediate should be seen with skepticism, when the proposed mechanism does not involve H-D exchange between D_2_O and a thiol.

**Figure 19:**
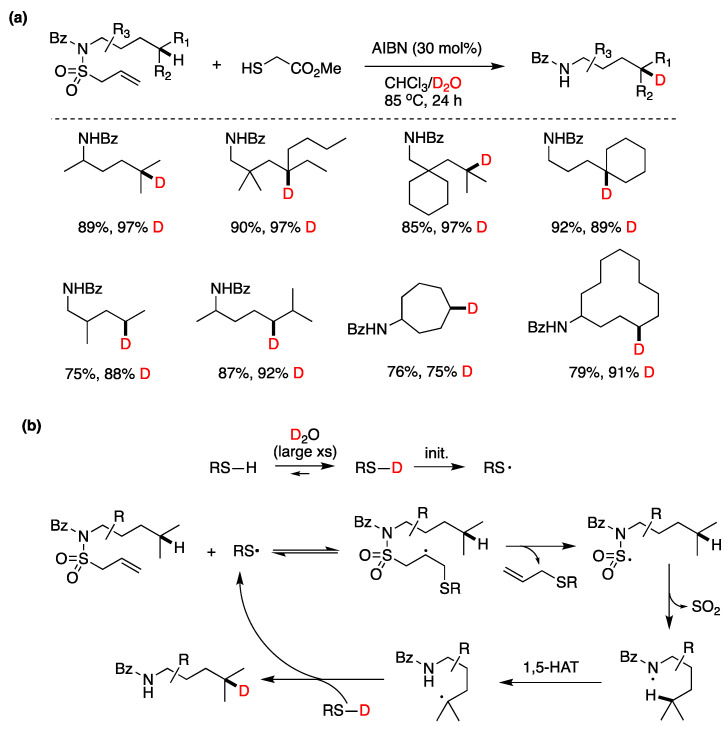
(**a**) Remote site-selective radical C(sp^3^)–H monodeuteration of amides and selected examples. (**b**) Proposed mechanism [93].

Although the assistance of thiols in radical reactions was well known, the in situ deuteration of thiols with D_2_O provides an easy and effective way to incorporate deuterium in stable C-D bonds, a process that finds applications in deuterium enrichment for the metabolic stabilization of drugs [105]. There are many applications that have been reviewed recently [106], but we can mention some notable recent examples that of course extend beyond the use of Et_3_B as an initiator. In recent work by the group of A. Studer [93], they applied the RSH/D_2_O protocol to an elegant remote site-selective radical C(sp^3^)–H monodeuteration of amides. In Figure 19, the proposed mechanism and scope of the reaction are described.

**Figure 20:**
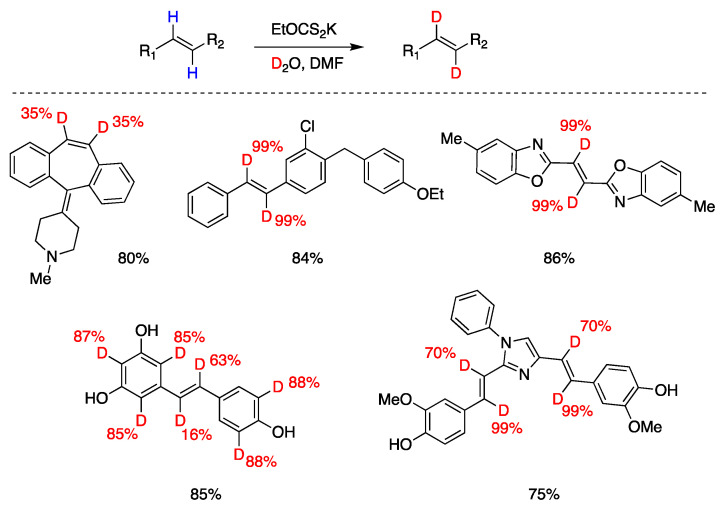
Synthesis of deuterated (*E*)-alkenes [107].

Q. Liu, Y. Li, and coworkers [107] developed a reversible, xanthate (EtOCS_2_K)-mediated, H-D exchange reaction of arene-conjugated (*E*)-alkenes using inexpensive D_2_O. The proposed mechanism involves the thermal generation of trisulfur radical anion (S_3_^•−^) from xanthate decomposition, which, after reversible addition–deuteration–elimination provides the deuterated products. Also, in this case, a xanthate S-D bond generated by reaction with D_2_O is responsible for the radical deuteration step. Complex substrates could be deuterated, and some polyphenols were also ortho-deuterated under the same conditions, although not consistently since in the examples presented some phenols are not ortho-deuterated (Figure 20).

Some recent publications that also fall under this category of thiol-assisted radical deuterations with D_2_O are described in the following section on photoredox processes [97,108,109].

### 5.2. Titanium Complexes

**Figure 21:**
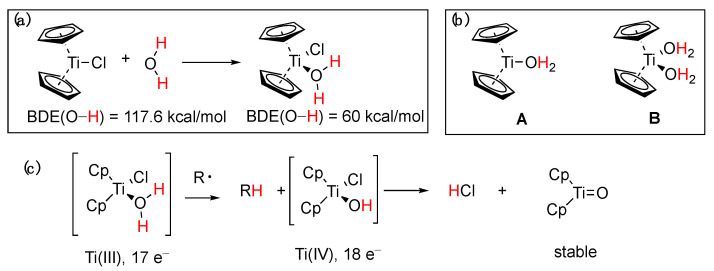
(**a**) Calculated BDE for the O-H bond of water complexed with Cp_2_Ti(III)Cl [88]; (**b**) active reductant forms according to ESR and theoretical calculations [112]; (**c**) proposed mechanism and intermediates [88].

**Figure 22:**
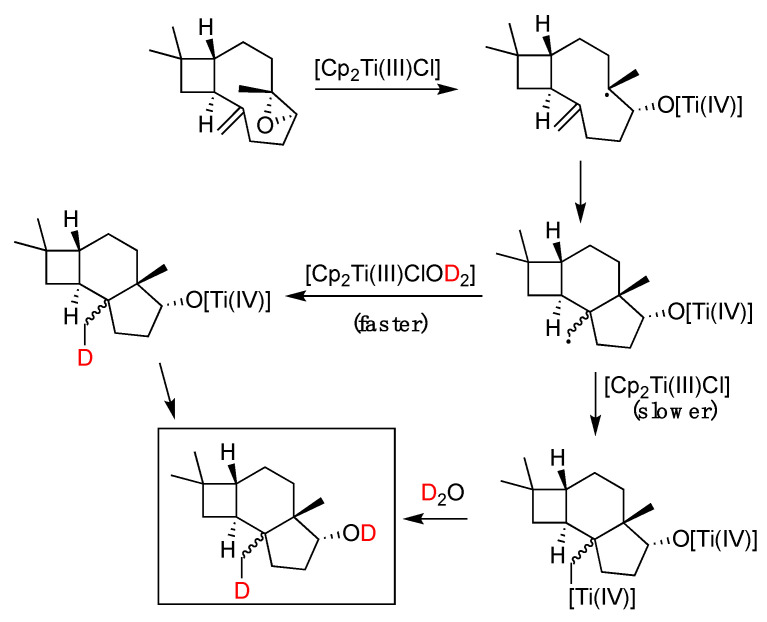
Proposed mechanism in a tricyclic system [114].

Around the same time that the boron-induced activation of water was first suggested, a similar observation was reported by J. M. Cuerva and coworkers [88] in the case of Ti(III). Specifically, it was proposed that aqua complexes of Cp_2_Ti(III)Cl (Nugent’s reagent) mediate the reductive opening of epoxides with a mechanism involving a HAT or DAT (deuterium atom transfer) from normal or heavy water to an intermediate free radical. A rate constant was later measured for the process [110], which was consistent with the role of water as the hydrogen atom donor, and a kinetic isotope effect k_H_/k_D_ of 3.35 was measured [88]. It was shown with UV–vis spectrophotometry that aliphatic and aromatic alcohols and amines exhibit a similar X-H bond weakening upon complexation with titanocene dichloride [111]. This particular reactivity of the Ti(III)–water complex was further studied with ESR and theoretical calculations, from which the active reductant forms of the titanocene–aqua complex were determined [112]. Theoretical calculations using DFT estimated a rather low BDE of 60 kcal/mol and a radical mechanism was proposed (Figure 21a–c). The proposed mechanism [88] involved competition between a slower anionic process and a faster radicaal process, as shown in Figure 22.

**Figure 23:**
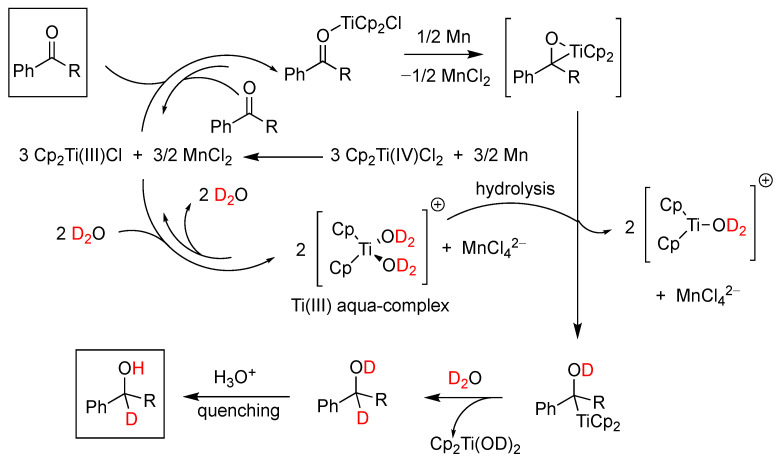
Revised mechanism for the Titanocene(III)/Mn-promoted reduction of ketones in aqueous media [114].

Synthetically, the Cp_2_Ti(III)Cl•D_2_O system has been utilized as a deuterating reductant in a series of transformations. In an interesting application [113], the system was applied to the reduction of propargyl halides, which through a proposed radical mechanism were transformed into exocyclic allenes. Similarly, A. Rosales et al. [114], in a mechanistic study of the titanocene(III)/Mn-promoted reduction of aromatic ketones in aqueous media, revised the previously proposed mechanism and proposed the intermediacy of a titanaoxirane (Figure 23) that in the presence of water undergoes hydrolysis towards the observed alcohol product. The authors cautioned that the free-radical character conventionally assumed for these chemical processes should be reconsidered.

### 5.3. Samarium(II) Iodide (SmI_2_)

**Figure 24:**
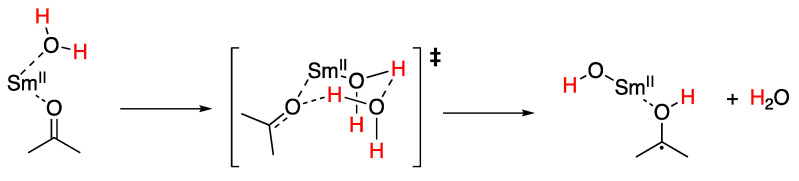
Proposed reduction of a ketone by Sm(II)I_2_–water proceeding through a highly ordered transition state.

Samarium(II) iodide (SmI_2_) is an important single-electron reductant in organic synthesis. Water, as a protic solvent additive, is of particular interest and has been used as a proton source in a wide range of reactions, including reductions and reductive coupling reactions. The reduction of trans-stilbene by SmI_2_–water allowed the determination of O–H bond weakening of at least 73.9 kcal/mol upon the coordination of water to SmI_2_ [115]. Flowers and co-workers found that the rates of reduction of a model aldehyde, ketone, and lactone were consistent with an initial proton-coupled electron transfer (PCET) from the SmI_2_–water reagent, proceeding through a highly ordered transition state shown in Figure 24 to form a ketyl radical intermediat [116].

**Figure 25:**
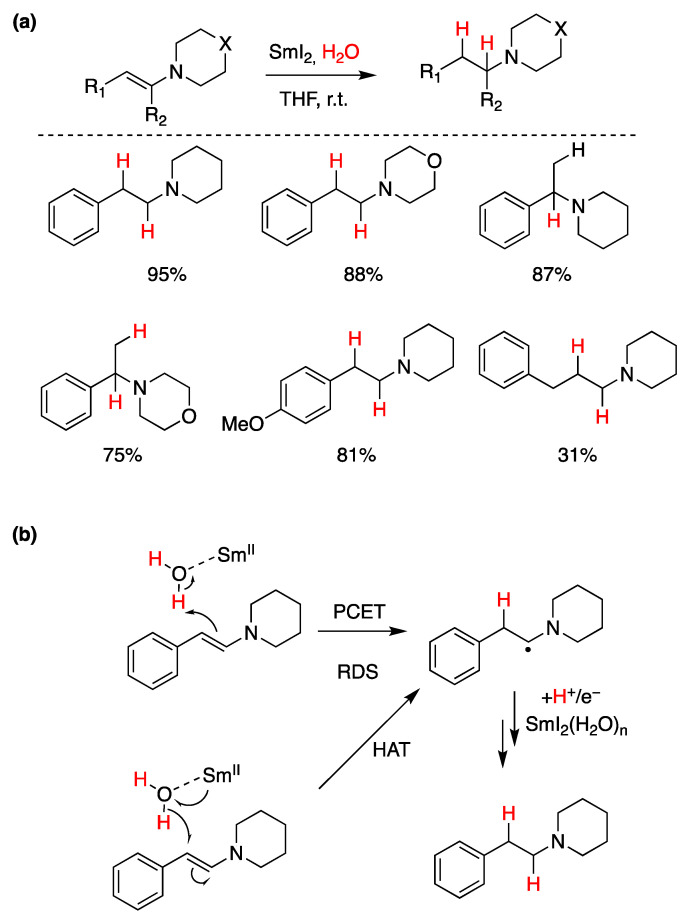
(**a**) Reaction scheme and substrate scope for the SmI_2_–water-mediated reduction of enamines. (**b**) Proposed PCET and HAT mechanisms for the reduction of enamines by SmI_2_-H_2_O [89].

The most common function of SmI_2_ is that of a reductant for oxygen-containing functional groups such as carbonyl-containing compounds. Recently, Mayer and co-workers found that samarium is significantly azaphilic and can reduce a range of tertiary enamines, as shown in Figure 25a [89]. Sequential ET-PT or PT-ET mechanisms were ruled out through competition reaction experiments. Instead, it was proposed that this reduction proceeds through either a concerted PCET or a formal hydrogen atom transfer (HAT), both illustrated in Figure 25b [89].

The O-H homolytic bond weakening of alcohols and water was estimated experimentally from the reduction of anthracene and benzyl chloride [116] but a poorly defined speciation of SmI_2_ in THF/alcohol mixtures limits reliable thermodynamic analyses of these systems. Recently, E.A. Boyd and J. C. Peters [117] utilized a bulky and strongly chelating ligand to facilitate clean electrochemical behavior. They revealed exceptionally weak N-H and O-H BDFEs of 27.2 and <24.1 kcal/mol, respectively, cementing the view that Sm(II) coordination induces the most significant bond weakening reported to date.

### 5.4. Molybdenum Complexes

**Figure 26:**
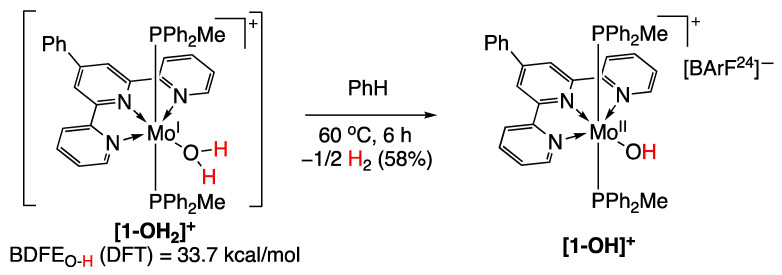
Spontaneous hydrogen evolution from a molybdenum Mo–aqua complex.

In 2016, the group of P. J. Chirik [95] reported on a number of molybdenum complexes with ammonia, hydrazine, or water that promoted hydrogen gas evolution due to a coordination-induced X–H (where X is nitrogen or oxygen) bond weakening. Calculations performed for the O–H bond of (^Ph^Tpy)(PPh_2_Me)_2_Mo(OH_2_)^+^ (Figure 26) determined a bond dissociation free energy (BDFE) value of 33.7 kcal/mol. The **[1-OH_2_]^+^** compound evolved hydrogen gas too rapidly to allow for characterization, leading to a more stable and isolable **[1-OH]^+^** compound (Figure 26) [95].

### 5.5. Germanium Corrole Complex

**Figure 27:**
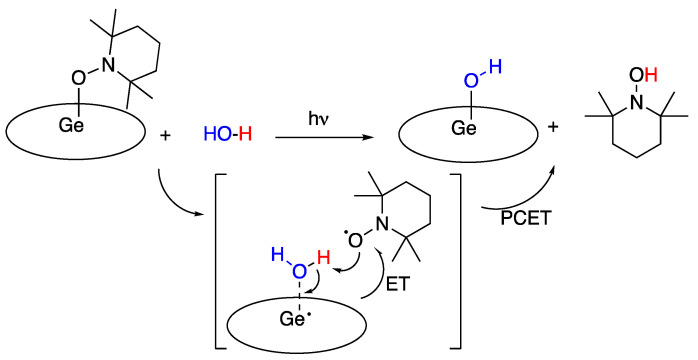
Reaction of a germanium corrole complex with water [94].

In 2015, X. Fu, S. Ye, and coworkers [94] reported a germanium (III) corrole complex, [(TPFC)Ge-(TEMPO)] (TPFC = tris(pentafluorophenyl)corrole, TEMPO = 2,2,6,6-tetramethylpiperidine-1-oxyl) that was found to react with water and produce (TPFC)Ge-OH and TEMPOH. Similar results were observed with methanol and ammonia, as well as primary aliphatic and aromatic and secondary aliphatic amines. Although the reaction proceeded slowly under thermal conditions (5 h) it was accelerated by visible light photolysis (1.4 h). The reaction was studied theoretically, and a mechanism was proposed, corroborated by DFT calculations (B3LYP and BP86), that involved water insertion between germanium and TEMPO, followed by a PCET rather than a HAT process, with an ET from the non-innocent corrole ligand to the TEMPO N–O bond, coupled with a proton transfer to TEMPO, as shown in Figure 27 below [94].

### 5.6. Organobismuth(II) Species

**Figure 28:**
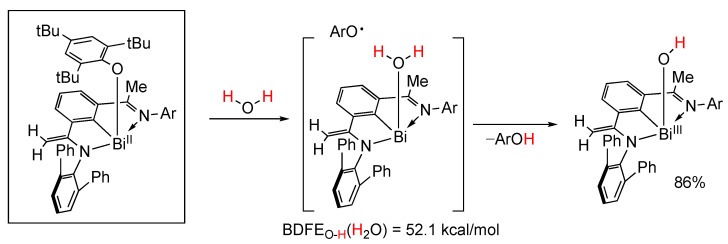
Reaction of H_2_O with a Bi(II) complex generates H_2_ and a Bi(III) hydroxy complex [96].

In a study similar to the above published in 2022, the group of J. Cornella reported on the radical activation of N–H and O–H bonds with bismuth [96]. They reported the synthesis and characterization of a radical equilibrium complex based on bismuth featuring an extremely weak Bi–O bond, which permitted the in situ generation of an organobismuth(II) species (Figure 28). As a result, radical activation of N–H and O–H bonds, including water, occurs in seconds at room temperature leading to a Bi(III) hydroxy complex. Theoretical calculations estimated a BDFE_O-H_(H_2_O) = 52.1 kcal/mol [96].

The recent work of A. Studer and coworkers [97] on an interesting photocatalytic system based on a phosphine-mediated water activation for radical hydrogenation, which will be described in the following section on photoredox processes, also falls within the area of coordination-induced bond weakening of O–H bonds, described in this section.

## 6. Photocatalysis in Water and Aqueous Media

The beginning of the era of photocatalytic reactions dates back to the 1900s, when Giacomo Ciamician clarified the influence of light in chemical reactions [118]. However, for nearly a century, photocatalysis received limited attention from researchers, probably due to the perception that controlling radical reactions was challenging. Based on pioneering studies by Kellogg and Deronzier, among others, it was only in 2008 that Macmillan [18], Yoon [119], and Stephenson [120] and their coworkers introduced the concept of photoredox catalysis. In this groundbreaking approach, transition-metal complexes were employed as photocatalysts, operating under mild reaction conditions, and demonstrating unprecedented utility across a range of chemical transformations. Since that pivotal moment, the field of photocatalysis has undergone substantial growth and diversification [121,122,123,124,125,126,127,128].

Recent applications of photoredox reactions using visible light have become increasingly popular not only because they operate under mild conditions but also because they eliminate the need for radical chemical initiators or the use of stoichiometric quantities of chemical reductants or oxidants in redox-neutral processes. The utilization of visible light as an irradiation source helps minimize waste and significantly reduces overall reaction costs. Photoredox catalysis also exhibits tolerance to various functional groups present in organic structures [124]. In a similar vein, the combination of water or water-based mixtures with organic solvents aligns with the principles of green chemistry, receiving support from both organic chemists and industry. In this context, harnessing the synergy between light and water presents exciting opportunities and expands the toolkit available to chemists for functionalization reactions driven by visible light photoredox catalysis. The appeal lies in the safety, affordability, and environmental friendliness of both water and visible light [9].

Most photocatalytic processes conducted in water are focused on eliminating contaminants, whether they are organic or organometallic in nature [129]. This is often achieved by harnessing the well-known redox properties of photocatalysts when they are in their excited states. These properties convert photocatalysts into highly oxidizing or reducing substances, creating reactive intermediates that facilitate the breakdown of contaminants. Importantly, this degradation occurs without the necessity of potent chemical oxidizers or reductants [130].

Nevertheless, the development of preparative organic photoredox catalytic methodologies in water or aqueous environments faces challenges, primarily due to the limited solubility of many organic substrates and catalysts in water or aqueous solvents. Additionally, conducting photoreactions in heterogeneous settings also presents difficulties. Recent review articles have focused on visible-light-mediated organic transformations in water or aqueous environments, placing particular emphasis on the choice of photocatalyst [131], the nature of the reaction medium [132], and the types of synthetic transformations achieved [133,134].

In this section, we will explore representative organic synthetic transformations towards the formation of C-C bonds, achieved through visible light photoredox catalysis in water or aqueous conditions, while also outlining the contributions by some of us in the field. Representative examples of radical additions to carbon–carbon multiple bonds and homolytic aromatic substitution reactions, including arylations and cyclization processes, will be discussed. Instances where water exerts significant influence on the outcomes of reactions will be presented. Water can also act as a proton or oxygen donor and may be necessary in stoichiometric quantities as a co-reactant in some cases. In other cases, water is utilized to dissolve specific reactants or photocatalysts. It is also adopted due to protocol improvements or more eco-friendly methods when contrasted with performing the same reaction in organic solvents. The overall objective of these various roles of water, whether as a reaction medium or merely as a component in photocatalytic processes, is to reduce the environmental consequences of both synthetic and photocatalytic procedures.

### 6.1. Photocatalyzed Radical Additions to Carbon–Carbon Multiple Bonds in Aqueous Media

Radical additions to carbon–carbon multiple bonds are very useful synthetic transformations, especially for producing medium-molecular-weight scaffolds from smaller molecules [135]. In this subsection, representative examples of radical alkylation [136], arylation [137], acylation [138], and fluoroalkylation [139], including atom transfer radical addition (ATRA) reactions [140] on carbon–carbon multiple bonds, will be presented.

**Figure 29:**
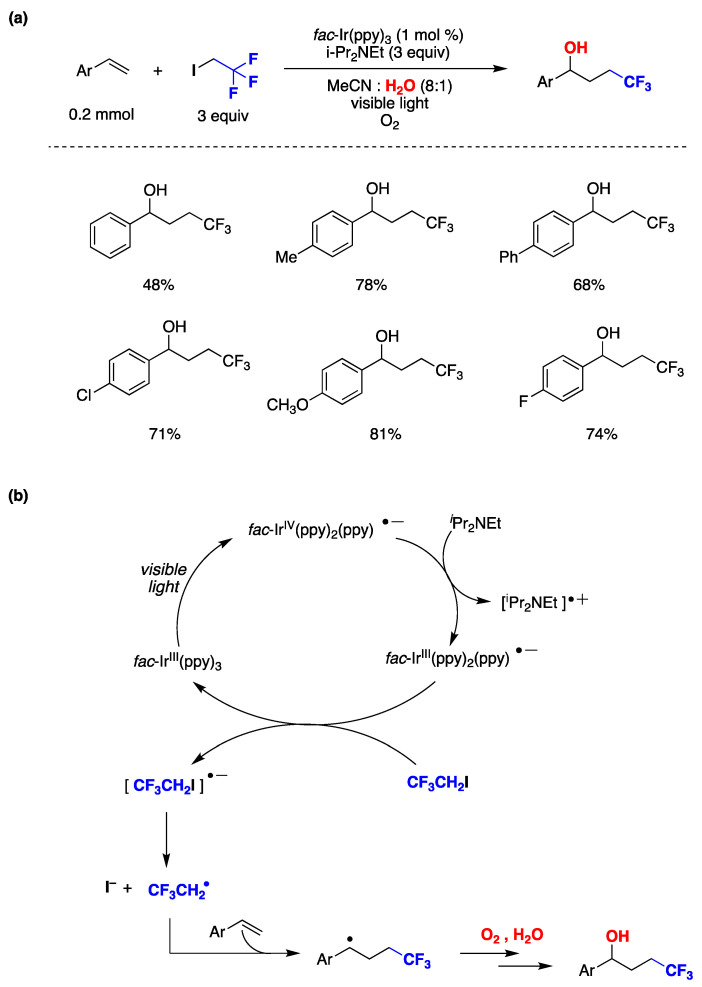
(**a**) Hydroxytrifluoroethylation of styrenes by photoredox catalysis in aqueous media and representative examples; (**b**) proposed reaction mechanism.

1,2-difunctionalization reactions of alkenes involving radicals are quite versatile, and various photocatalytic techniques have been utilized for this purpose [141]. Chen, Guo, and collaborators developed a novel methodology involving photocatalysis and the use of water as a co-solvent to achieve hydroxytrifluoroethylation of styrenes [142]. In a standard procedure, the authors employed fac-Ir(ppy)_3_ as a photoredox catalyst, and *N*,*N*-diisopropylethylamine as a reductive sacrificial donor in acetonitrile:water (8:1) as reaction media under an oxygen atmosphere. The presence of water played a pivotal role in the outcome of the reaction, as the desired product failed to form without it. The authors explored various styrene derivatives, including those with electron-donating or halogen substituents, and found that the reaction proceeded smoothly in all cases, as shown in Figure 29a. Based on different mechanistic studies, the authors proposed a reaction mechanism (Figure 29b) where the photocatalyst *fac*-Ir^III^(ppy)_3_ is initially promoted to its excited state and further reduced by *N*,*N*-diisopropylethylamine, generating *fac*-Ir^III^(ppy)_2_(ppy)^•−^. A CF_3_CH_2_I molecule is reduced by *fac*-Ir^III^(ppy)_2_(ppy)^•−^ regenerating the photocatalyst and affording I^−^ and a trifluoroethyl radical (CF_3_CH_2_^•^). The CF_3_CH_2_^•^ radical is subsequently captured by the styrene derivative, generating a benzyl-type radical. In the presence of oxygen and water, this radical reacts with molecular oxygen leading to the production of the hydroxytrifluoroethylation reaction product (Figure 29b). Water plays a crucial role in the reaction, as its absence leads to the formation of 2,2,2-trifluoroethanol as the product, with no observation of the difunctionalized product. Furthermore, an experiment using ^18^O_2_ or H_2_^18^O suggested that the oxygen in the product comes from molecular oxygen present in the reaction media and not from water [142].

**Figure 30:**
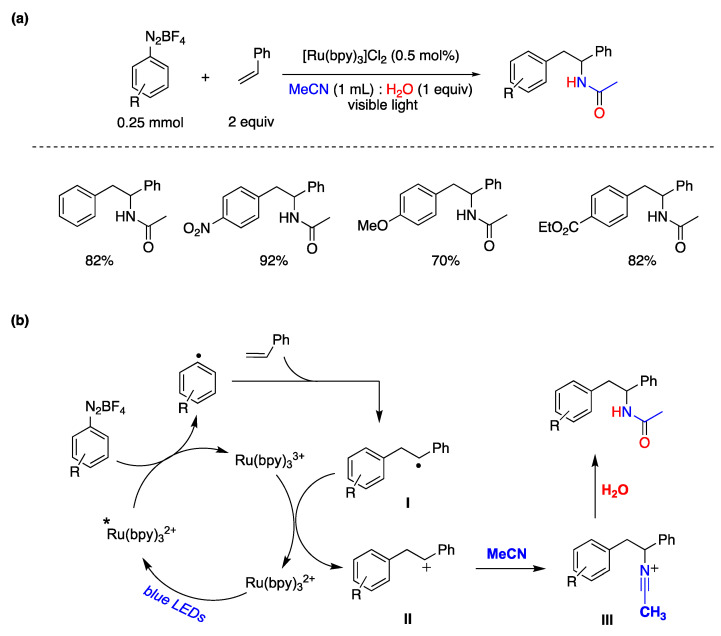
(**a**) Arylation of styrene by photoredox catalysis in MeCN:H_2_O as reaction media and representative examples; (**b**) proposed reaction mechanism.

Ever since the Meerwein reaction was initially introduced, aryl radicals have demonstrated their versatility as intermediates in organic synthesis, especially in processes involving functional group interconversions and the formation of C–C bonds [134,143,144]. In 2014, König and colleagues reported a photocatalytic arylation of styrene derivatives using aryldiazonium salts as substrates [145]. They employed [Ru(bpy)_3_]Cl_2_ as the photocatalyst and irradiated the reaction with a 440 nm light source in a reaction medium consisting of CH_3_CN and water. The versatility of this transformation with various aryl diazonium salts is outlined in Figure 30a. The proposed mechanism is based on experimental evidence and prior research findings, as depicted in Figure 30b. Initially, an aryl radical is generated through electron transfer from the excited state of the photocatalyst [Ru(bpy)_3_]^2+^* to the diazonium salt generating [Ru(bpy)_3_]^3+^. The aryl radical adds to the olefin, forming radical intermediate **I**, which is subsequently oxidized by Ru(bpy)^3^]^3+^ to produce carbocation **II** and regenerate the photocatalyst. Finally, intermediate **II** reacts with the solvent (acetonitrile), leading to the formation of intermediate **III**, followed by hydrolysis to yield the amide product through a Ritter-type process. The involvement of water in the reaction is essential as it provides the hydrogen and oxygen atoms necessary for the attack by water in the transformation of intermediate **III** to the final reaction product (Figure 30) [145].

Ketones are versatile building blocks in organic synthesis due to their inherent ability to participate in a wide array of bond-forming reactions as electrophilic compounds. As a result, the synthesis of ketone groups has been a focus of extensive research for many years. Traditional approaches for generating ketones can be broadly categorized into the following four groups, depending on the active intermediate derived from the starting materials: (1) the oxidation of alcohols, (2) the acylation of carbon-centered nucleophiles, (3) the addition of acyl radicals to unsaturated moieties, and (4) the acylation of carbon-centered radicals [138]. Indeed, recent decades have been a flourishing period in the field of organic radical chemistry that delivered ground-breaking results, especially in organic synthesis. Consequently, a diverse array of radical acylation reagents and catalytic systems have been meticulously crafted and advanced, following the publication of a seminal review on radical acylation authored by S. Kim and colleagues in 2004 [146].

**Figure 31:**
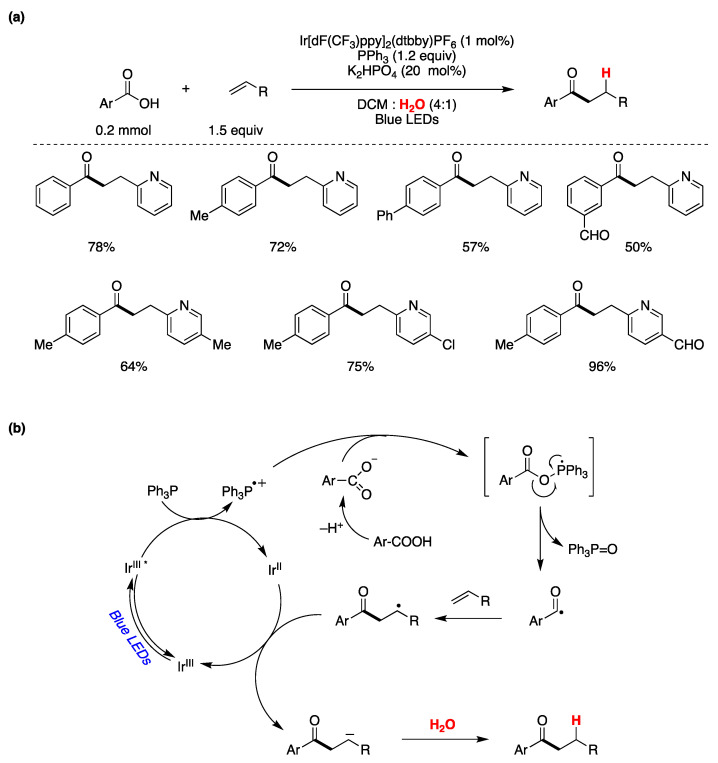
(**a**) Synthesis of aromatic ketones by deoxygenative carbon–carbon coupling of aryl-carboxylic acids with olefins in an aqueous phase and representative examples; (**b**) proposed reaction mechanism.

Zhang, Xie, and Zhu have presented a visible-light-mediated photocatalytic process that involves the deoxygenation of aryl carboxylic acids facilitated by PPh_3_ for the generation of acyl radicals [147]. This transformation results in the deoxygenative coupling of aryl carboxylic acids with olefins, occurring in an aqueous environment to yield aromatic ketones. The authors determined the optimal reaction conditions as those shown in Figure 31a. They also explored the reaction’s applicability with various aryl carboxylic acids and alkenes, and the outcomes of this exploration are summarized in Figure 31a. After conducting mechanistic investigations, the authors proposed a plausible reaction mechanism, as depicted in Figure 31b. Upon visible light excitation, the photocatalyst in its excited state oxidizes Ph_3_P to its radical cation, while returning to its ground oxidation state. The resulting Ph_3_P^·+^ species combines with the aryl carboxylate ion, forming a P-centered radical. This process triggers the cleavage of the O–C=O bond, yielding an acyl radical and Ph_3_P=O. The acyl radical subsequently attacks the double bond of the olefin, resulting in the formation of a radical adduct. This adduct is further reduced by the lower oxidation state of the photocatalyst, generating a carbanion. Upon protonation from water, the carbanion ultimately produces the final reaction product (Figure 31b). Deuterium labeling experiments conducted in D_2_O revealed that water served as the proton source. When the aromatic acid was labeled with ^18^O, Ph_3_P=^18^O was formed, indicating that the oxygen atom in Ph_3_P=^18^O originated from the carboxylic acid and not from water [147].

**Figure 32:**
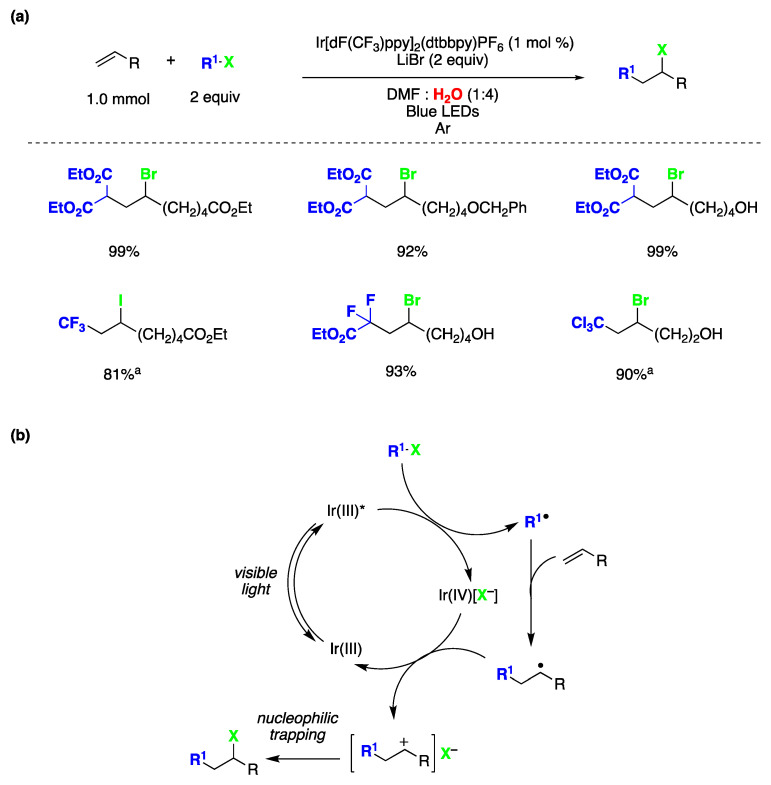
(**a**) Intermolecular atom transfer radical addition (ATRA) to olefins by photoredox catalysis in aqueous media and representative examples, (^a^) without LiBr; (**b**) proposed reaction mechanism.

Atom transfer radical addition (ATRA) reactions are pivotal processes in synthetic chemistry, allowing for the efficient dual functionalization of alkenes while maximizing the atom economy [140]. Stephenson and coworkers presented a seminal study on photocatalyzed ATRA reactions conducted in an aqueous environment [148]. This publication marks the first documented instance of intermolecular ATRA reactions involving haloalkanes and α-halocarbonyls with olefins, all facilitated by photoredox catalysis activated by visible light in aqueous media. The optimal reaction conditions for this transformation are depicted in Figure 32a. The versatility of this reaction was demonstrated with various olefins featuring diverse functional groups, including alcohols, benzyl esters, alkyl bromides, silyloxy esters, esters, enones, carbamates, and aromatic rings. Regarding the atom transfer agent, the reaction exhibited good compatibility with a range of haloalkanes and α-halocarbonyls, including CF_3_I (Figure 32a). Taking into consideration multiple mechanistic studies, the authors proposed the reaction mechanism depicted in Figure 32b. The reaction commences with photoexcitation of the Ir(III) photocatalyst, resulting in the formation of an excited species of Ir(III)*, which undergoes a single-electron transfer process with the haloalkane (or α-halocarbonyl), leading to the generation of Ir(IV) (with a halide as a counterion) and the electrophilic alkyl radical. The alkyl radical subsequently adds to the olefin to form the radical adduct. The formation of the ATRA reaction product can occur through oxidation of the radical adduct by Ir(IV) associated with the halide counterion, resulting in the formation of a carbocation. This carbocation then rapidly reacts with the halide anion to yield the desired reaction product. It is important to note that external nucleophiles, including water (which serves as a major co-solvent), do not participate in reactions with the proposed carbocation intermediate, as evidenced by mechanistic experiments conducted by the authors [148].

**Figure 33:**
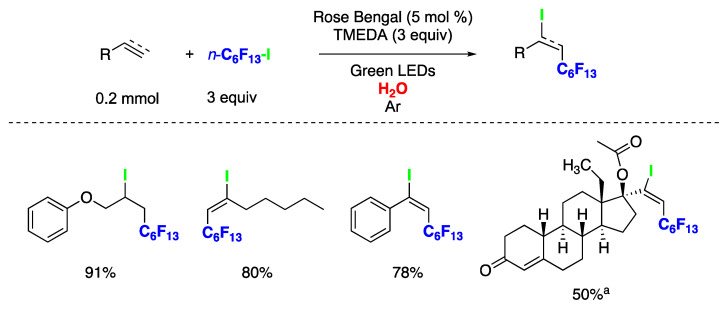
Representative examples for the photocatalyzed iodoperfluorohexylation of olefins and alkynes in water; (^a^) in MeOH:H_2_O (1:2).

The iodoperfluorohexylation of olefins and alkynes in water has been recently reported [149]. These represent the first examples of photocatalyzed ATRA reactions conducted entirely in water as the solvent. Optimized reaction conditions are shown in Figure 33. Both alkenes and alkynes rendered products derived from the ATRA pathway, and in the case of alkynes, exclusively as *E*-stereoisomers (Figure 33). This synthetic approach was also applied to the late-stage functionalization of the pharmacologically active alkyne drug (D)-(−)-Norgestrel acetate, a hormonal medication and contraceptive, with a 50% yield using the aqueous medium MeOH:H_2_O (1:2). Mechanistic investigations provided evidence for the involvement of a radical pathway. However, the authors were unable to conduct optical experiments to assess the operation of an oxidative or reductive photocatalytic quenching cycle [149]. This limitation stems from the poor solubility of C_6_F_13_-I in the reaction medium, preventing the measurement of reliable optical spectroscopy values for Stern–Volmer kinetic analysis or triplet quenching experiments.

**Figure 34:**
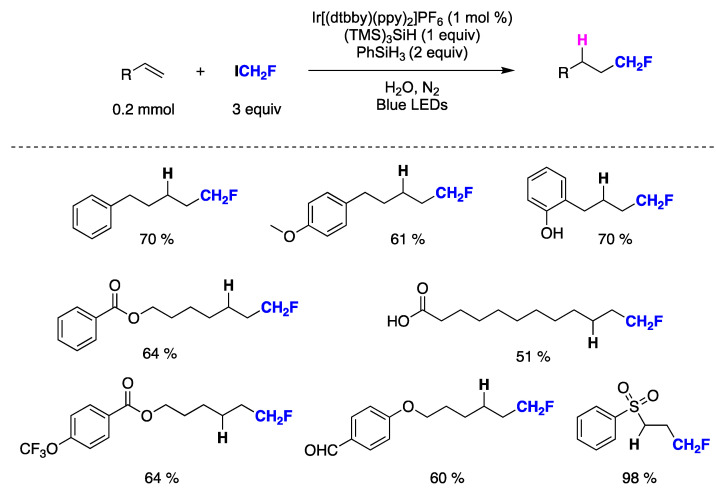
Photoredox-catalyzed and silane-mediated hydrofluoromethylation of unactivated alkenes in water, and representative examples.

Qing and collaborators have recently developed the first methodology for the hydrofluoromethylation of unactivated alkenes [150]. This innovative method utilizes fluoroiodomethane and hydrosilanes, merging photoredox catalysis with silane-mediated deiodination processes. Key aspects of the procedure involve the utilization of water as the solvent, ICH_2_F for CH_2_F radical generation, PhSiH_3_ as the H-donor, and (TMS)_3_SiH as an additive to achieve higher yields. The mild reaction conditions enable the tolerance of various functional groups such as phenol, ether, aldehyde, carboxylic acid, ester, sulfone, trifluoromethyl, and trifluoromethoxy, among others (Figure 34). Initial mechanistic investigations suggest that employing water as the solvent facilitates the addition of CH_2_F radicals to unactivated alkenes, and the incorporation of (TMS)_3_SiH significantly enhances chemoselectivity.

**Figure 35:**
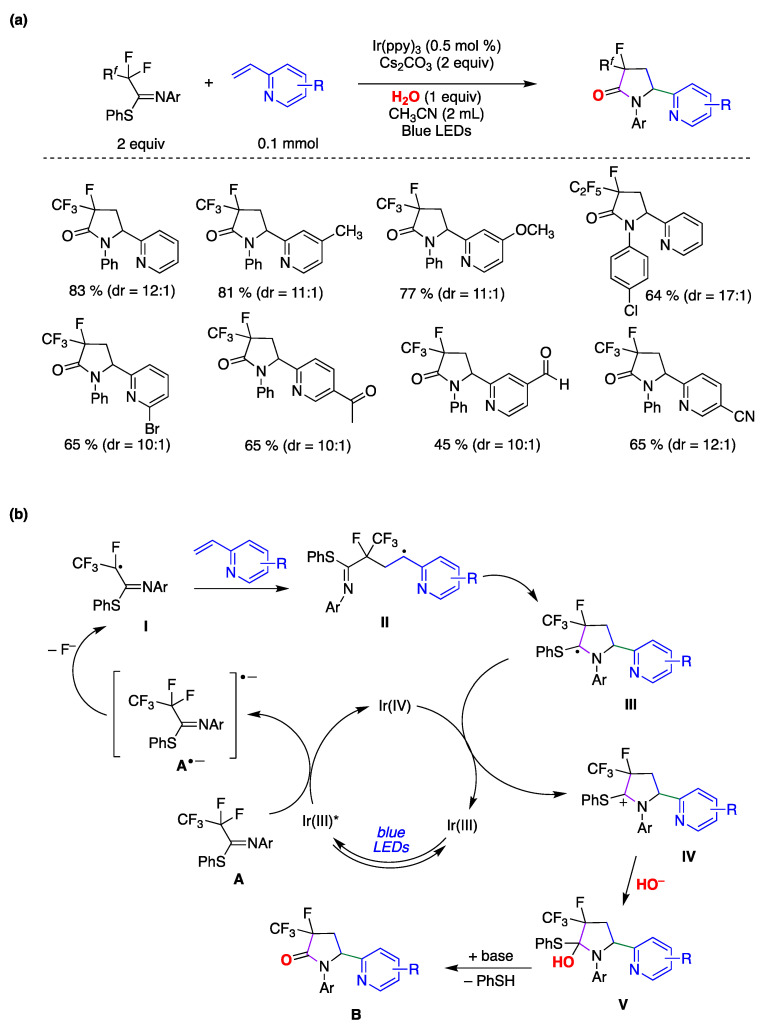
(**a**) Photocatalytic single C(sp^3^)–F bond activation of perfluoroalkyl iminosulfides with alkenes in water and selected examples; (**b**) proposed reaction mechanism.

Liu and colleagues reported on a direct and site-specific C(sp^3^)–F bond alkylation in polyfluorinated iminosulfides using alkenes and water through photoredox catalysis, yielding a diverse range of 3-fluoro-3-perfluoroalkyl-γ-lactams, accompanied by the simultaneous formation of C(sp^3^)–C(sp^3^), C(sp^3^)–N bond, and C=O bonds (Figure 35) [151]. The study revealed that various substituted 2-vinylpyridines effectively participated in the reaction, producing the corresponding products in moderate to high yields and high to excellent diastereoselectivities. The conditions proved compatible with diverse functional groups and substituents, such as methyl, bromo, chloro, ketone, ester, aldehyde, methoxyl, and cyano, among others (Figure 35a). Notably, a range of perfluoroalkyl units (R*^f^*), including C_2_F_5_, C_3_F_7_, C_4_F_9_, and C_5_F_11_, underwent site-specific defluorofunctionalization. This approach demonstrated precise chemoselectivity control and exhibited outstanding tolerance toward various functional groups. Considering several mechanistic studies, the authors proposed a plausible reaction mechanism outlined in Figure 35b [151]. Initially, the Ir(III) photocatalyst, upon irradiation, is promoted to its excited state, Ir(III)*. Subsequently, Ir(III)* reduces **A** through single-electron transfer (SET), producing the Ir(IV) species and radical anion A^•−^. Following this, a spin-center shift process takes place, leading to the formation of the corresponding radical **I** with the cleavage of a C–F bond. **I** is captured by 2-vinylpyridine derivative to form the alkyl radical intermediate **II**. This intermediate is then intercepted by the C=N double bond of the imine group via a 5-endo-trig cyclization, resulting in the formation of the C-centered radical intermediate **III**. The photoredox cycle is subsequently closed by SET between **III** and Ir(IV), giving rise to the carbocationic intermediate **IV**, which is then trapped by the hydroxyl anion, yielding intermediate **V**. Finally, **V** undergoes elimination, prompted by the base, resulting in the formation of a polyfluorinated γ-lactam **B** and thiophenol. It must be pointed out that by ^18^O-labeling experiments the authors confirmed that the oxygen atom in the amide group originates from water.

### 6.2. Photocatalyzed Homolytic Aromatic Substitutions in Aqueous Media

Homolytic aromatic substitution (HAS) is a practical synthetic approach used to exchange aromatic hydrogen atoms with appropriate substituents, facilitating the formation of new C–C or C–heteroatom bonds. Currently, due to the advances in the field of synthetic radical organic chemistry, HAS has become the preferred synthetic approach, competing with organometallic, transition-metal, and electrophilic aromatic substitution methodologies. In the last decade, visible-light photoredox catalysis has emerged as a pivotal approach for aromatic substitution. The main photoredox catalysts employed include polypyridine complexes of Ru(II) and Ir(III), as well as organic photoredox catalysts, providing a metal-free option for HAS [152].

**Figure 36:**
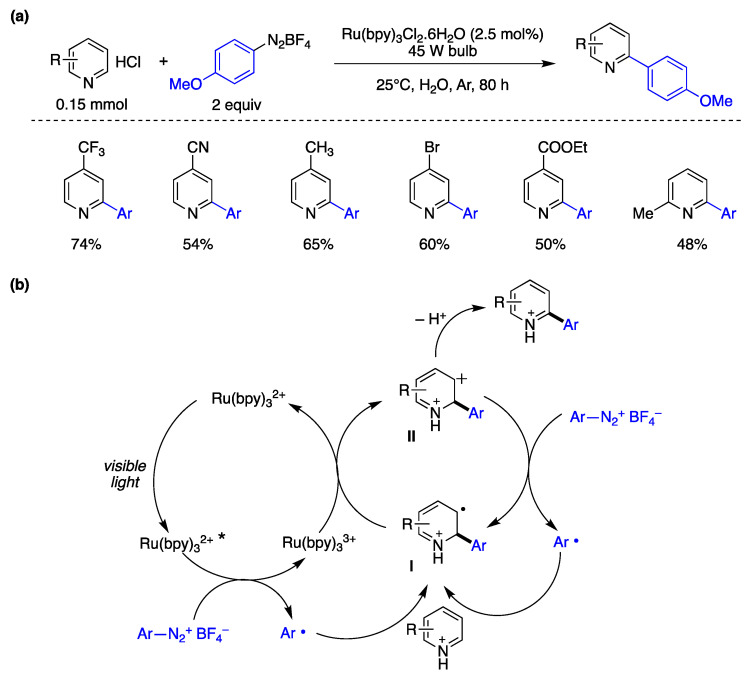
(**a**) Photocatalyzed arylation of pyridine hydrochloride derivatives in water and representative examples; (**b**) proposed reaction mechanism.

Xue, Xiao, and collaborators have introduced a photocatalytic arylation methodology for electron-deficient heteroarenes in water as the solvent [153]. As depicted in Figure 36a, pyridines featuring electron-withdrawing groups (such as CF_3_, CN, COOEt, and Br) or electron-donating groups (such as CH_3_) resulted in reasonably good yields of arylated products. The authors subsequently examined the substrate scope by evaluating different aryl diazonium salts, functionalized with groups such as cyano, carboxyethyl, chlorine, bromine, and fluorine, as aryl radical precursors. In these studies, the authors utilized 4-trifluoromethylpyridine hydrochloride as the radical acceptor, successfully yielding the corresponding arylation products in satisfactory yields. Furthermore, the authors explored a one-pot approach by synthesizing the aryldiazonium salt and conducting the in situ arylation of the heteroaromatic compound in water. This approach also yielded reasonable yields of the coupling products [153]. Based on mechanistic experiments performed and established literature precedents, the authors proposed the mechanism illustrated in Figure 36b. The process initiates with the excitation of the Ru(bpy)_3_^2+^ photocatalyst (Scheme X11) upon exposure to visible light, resulting in the formation of [Ru(bpy)_3_^2+^]*. A reductive electron transfer process between [Ru(bpy)_3_^2+^]* and the aryldiazonium salt generates an aryl radical Ar^•^. This aryl radical engages a homolytic aromatic substitution process with a pyridinium chloride, leading to the formation of radical intermediate **I**. Subsequent oxidation by an additional aryldiazonium salt species generates the cationic intermediate **II** and aryl radicals, which then propagate the chain reaction. Deprotonation of intermediate **II** ultimately yields the arylated pyridine product. Although this reaction appears straightforward and has been conducted in various organic solvents under different radical conditions, the authors’ enhanced methodology, employing water as the reaction medium and utilizing photocatalysis, offers distinct advantages. Notably, the use of water has improved the reactivity of pyridine nuclei as pyridinium salts in arylation reactions, resulting in advantages such as enhanced regioselectivity (substitution of pyridine rings at the 2-position), utilization of aryl precursors from soluble benzenediazonium salts, and improved arylation product yields [153].

**Figure 37:**
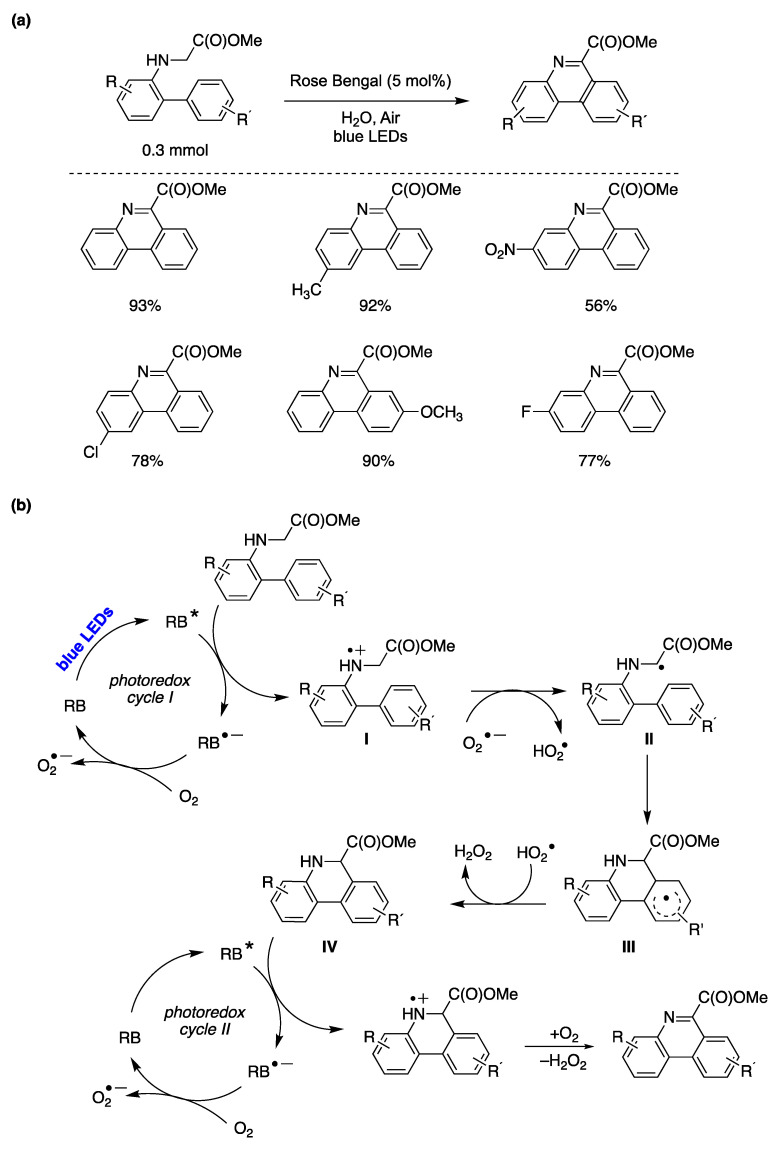
(**a**) Photocatalyzed synthesis of phenanthridine-6-carboxylates from *N*-biarylglycine esters in water and representative examples; (**b**) proposed reaction mechanism.

Natarajan and colleagues successfully developed a protocol for synthesizing phenanthridine-6-carboxylates from *N*-biarylglycine esters in water as the solvent [154]. The authors determined the optimal reaction conditions as those shown in Figure 37a. To explore the substrate scope for this transformation, the authors examined various *N*-biarylglycine methyl esters with different substituents on the aromatic rings (Figure 37a). As shown in the figure, both electron-withdrawing and electron-donating substituents on the biaryl moiety resulted in high yields of substituted phenanthridines. The authors proposed a reaction mechanism for the synthesis of phenanthridine-6-carboxylates from *N*-biarylglycine esters, as illustrated in Figure 37b. Initially, the excited photocatalyst Rose Bengal (RB*) oxidizes the *N*-biarylglycine ester substrate to form radical cation **I**, concomitantly generating the radical anion of RB^•−^, which then reduces the oxygen present in the reaction medium to a superoxide anion regenerating the photocatalyst. The superoxide anion subsequently deprotonates intermediate radical cation I, yielding radical II and a hydroperoxyl radical. Through a HAS process, radical II produces cyclohexadienyl radical intermediate III. The hydroperoxyl radical previously formed abstracts a hydrogen atom from intermediate III, yielding dihydrophenanthridine IV. In a second catalytic cycle, the oxidation of IV occurs, ultimately yielding the phenanthridine reaction product. Independent experiments conducted with dihydrophenanthridine IV under irradiation with Rose Bengal as the photocatalyst, using the optimized reaction conditions, resulted in the quantitative yield of the phenanthridine product. This demonstrates the effective oxidation of dihydrophenanthridine IV within the photocatalytic cycle to yield the phenanthridine final reaction product (Figure 37) [154].

**Figure 38:**
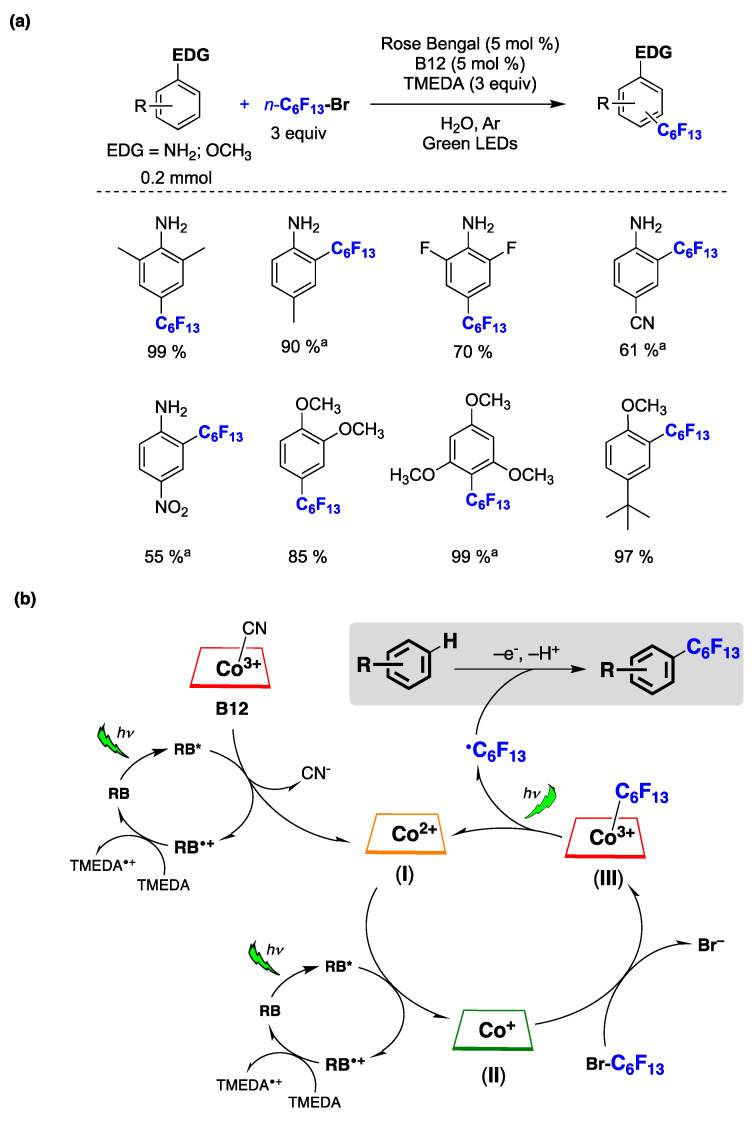
(**a**) Photocatalyzed C-H fluoroalkylation of arenes in water promoted by vitamin B12 and Rose Bengal, and representative examples; (^a^) water:acetonitrile (1:1); (**b**) proposed reaction mechanism.

Innovative synthetic techniques for producing fluoroalkylated aromatic compounds are in great demand because of their distinctive characteristics, which enable their use in various fields, such as medicinal chemistry, agrochemistry, and materials science. Within this context, radical fluoroalkylation reactions mediated by catalytic cycles driven by light have gained significant attention over the past decade [155]. The first perfluoroalkylation reaction of activated arenes in water has recently been reported [156]. Optimized reaction conditions are shown in Figure 38a. The reaction scope was extended to different amino-substituted arenes and alkoxyarenes bearing electron-donating or electron-withdrawing groups, yielding the corresponding perfluoroalkyl-substituted products in good to excellent yields (Figure 38a) [156]. The authors, based on the mechanistic investigations performed and information available in the literature, proposed a plausible reaction mechanism, outlined in Figure 38b [156]. The sequence begins with vitamin B12 undergoing a one-electron reduction process facilitated by a Rose Bengal (RB) oxidative photocatalytic cycle. This reduction leads to the formation of cob(II)alamin I upon cyanide loss. The reaction proceeds with the further reduction of I via an additional RB oxidative photocatalytic cycle, resulting in the generation of cob(I)alamin II, which rapidly reacts with *n*-C_6_F_13_Br, producing the Co(III)-C_6_F_13_ complex III and a bromide anion. Upon exposure to light, the complex III releases an *n*-C_6_F_13_^•^ radical and regenerates I, thereby completing the cobalt-mediated co-catalytic cycle (Figure 38b). The *n*-C_6_F_13_^•^ radical formed reacts with the arene via a HAS mechanism affording the perfluorohexylated reaction product.

**Figure 39:**
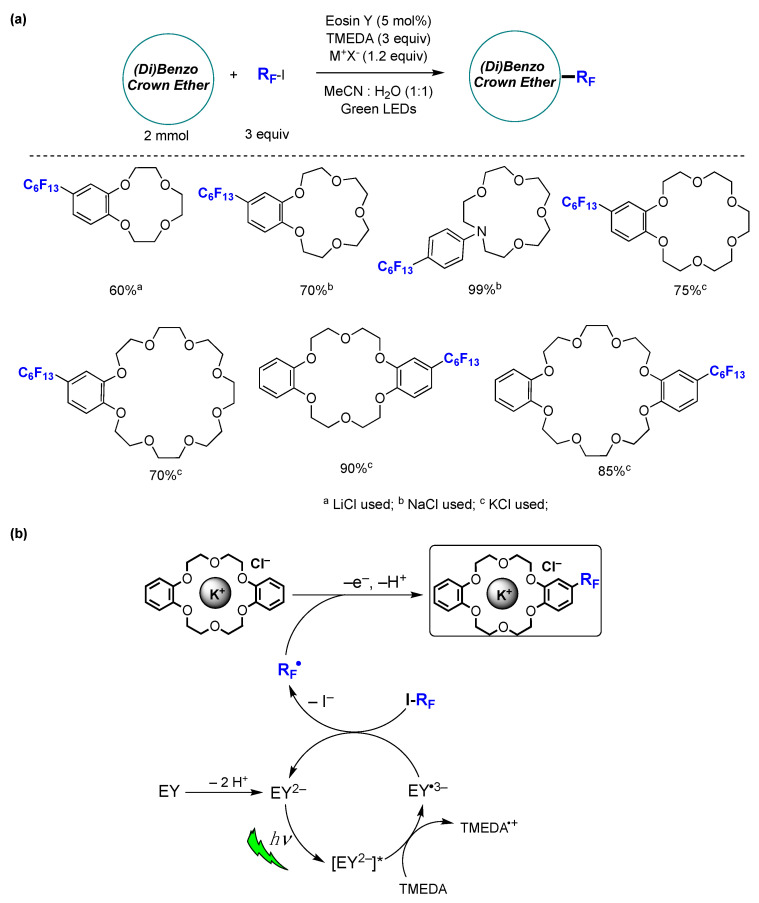
(**a**) Late-stage photocatalytic fluoroalkylation of aromatic crown ethers in aqueous media and representative examples; (**b**) proposed reaction mechanism.

Modified crown ethers are essential building blocks in supramolecular chemistry, finding uses in phase transfer catalysis, metal extraction, intelligent materials, and molecular machines [157]. A successful protocol for the late-stage incorporation of fluoroalkyl moieties into (di)benzo crown ethers has been reported [158]. The photocatalyzed reactions were carried out in mixed aqueous–organic solvents, as the inclusion of water was found to be essential for maximizing the yield. The optimized reaction conditions achieved are shown in Figure 39a. The capacity of crown ethers to form complexes with metal ions played a crucial role in enhancing the solubility of the substrates within the reaction mixture. This, in turn, facilitated perfluoroalkyl group substitution with high yields, along with remarkable chemo- and regioselectivity. Different fluoroalkyl-substituted crown ethers were prepared and isolated in yields ranging from 60 to 99%, proving the wide scope of the protocol. The authors also showed that a (di)benzo crown ether within a complex rotaxane structure can efficiently undergo direct perfluoroalkylation with high chemo- and regioselectivity using the described method. This underscores the ability of the methodology to incorporate stereogenic and/or functional elements into mechanically interlocked structures with remarkable ease and efficiency [158]. Based on mechanistic investigations and information available in the literature, the authors have put forth a plausible reaction mechanism, illustrated in Figure 39b. Under the studied reaction conditions, EY, which contains two relatively acidic protons (pKa 2.0, 3.8, in water), readily undergoes deprotonation by TMEDA, leading to the quantitative formation of EY^2−^. In contrast to EY, EY^2−^ exhibits strong absorption in the green region of the UV–vis spectra. Upon green light irradiation, the triplet state is typically considered the most relevant excited state for EY^2−^ due to its very brief singlet lifetime. Furthermore, the ^3^[EY^2−^]* state can serve as both a moderate oxidant and reductant. It was suggested that, under the investigated reaction conditions, EY^2−^ functions as an oxidant by accepting an electron from TMEDA. This is supported by a favorable spontaneous electron transfer, indicated by a ΔG_PET_ value of −0.36 eV. Subsequently, perfluoroalkyl radicals (R_F_^•^) could be generated by the reaction of EY^•3−^ with the corresponding perfluoroalkyl iodides (R_F_I). The R_F_^•^ radical formed then reacts with the (di)benzo crown ether metal cation complex through a homolytic aromatic substitution mechanism, resulting in the formation of the perfluorohexylated reaction product (Figure 39b) [158].

### 6.3. Miscellaneous

**Figure 40:**
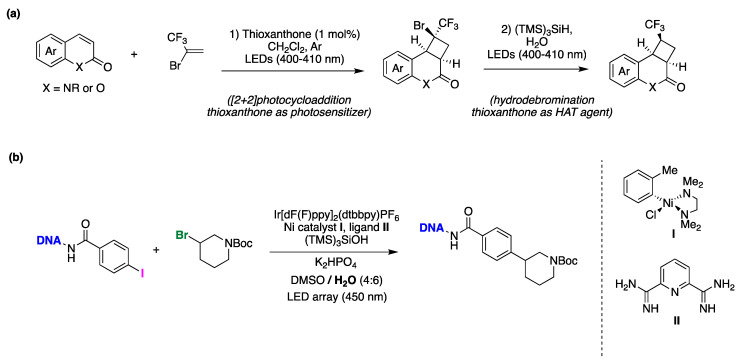
(**a**) Diastereoselective synthesis of trifluoromethylated cyclobutane derivatives by [2+2]-photocycloaddition followed by water-assisted hydrodebromination. (**b**) Photoredox cross-electrophile coupling of alkyl bromides with DNA-tagged aryl iodides in aqueous solution.

Guo, Liu, and collaborators [159] recently reported a method for the diastereoselective synthesis of trifluoromethylated cyclobutane derivatives under visible light irradiation. This approach involves a one-pot process that combines [2+2]-photocycloaddition with water-assisted hydrodebromination (Figure 40a). Various compounds, including quinolinones, isoquinolinones, and coumarins, can successfully participate in this one-pot process with 1-bromo-1-trifluoromethylethene. Furthermore, stereo-defined trisubstituted trifluoromethylated cyclobutane alcohols, carboxylic acids, and amines can be straightforwardly obtained by the ring opening of lactone or lactam, maintaining the original high diastereoselectivity facilitated by water-tristrimethylsilylsilane coordination. Thioxanthone (TX) functioned as both a photosensitizer and a hydrogen atom transfer (HAT) agent. The diastereoselectivity in the hydrogen atom abstraction process was regulated by (TMS)_3_SiH and assisted by water. This approach served as a complementary method to achieve a novel mode of hydrodebromination under mild conditions. TX played a pivotal role in facilitating this one-pot transformation, acting as a suitable substitute for transition-metal and radical initiators [159].

Kölmel, Ratnayake, and Flanagan [160] developed a catalytic process that allows the photoredox cross-electrophile coupling of alkyl bromides with DNA-tagged aryl iodides in an aqueous environment (Figure 40b). The success of this metallaphotoredox transformation relies on the utilization of novel pyridyl bis(carboxamidine) ligands crucial to the nickel catalytic cycle (see the right part of Figure 40b for chemical structures). The described C(sp^2^)–C(sp^3^) coupling exhibits broad tolerance for both DNA-tagged aryl iodides and alkyl bromides. Significantly, the reaction has been optimized for parallel synthesis, a key requirement for the efficient preparation of combinatorial libraries, utilizing a 96-well-plate-compatible blue LED array as the light source. Consequently, this mild and DNA-compatible transformation is well-suited for the construction of DNA-encoded libraries.

**Figure 41:**
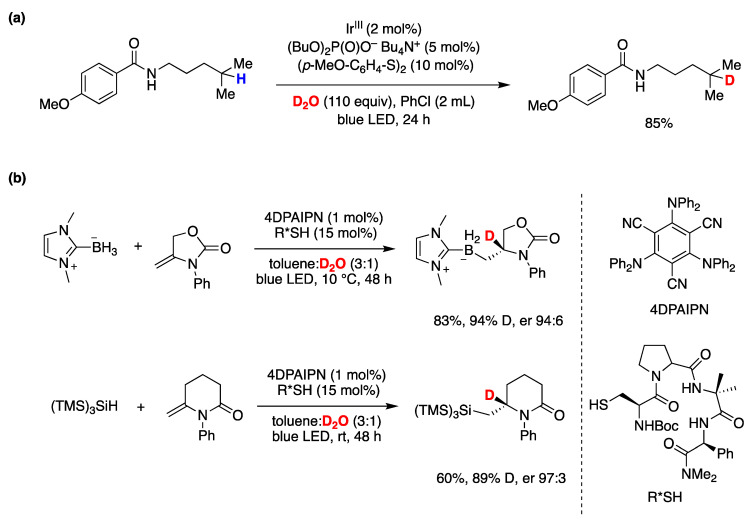
Photoredox catalysis for radical deuteration: (**a**) an example of H/D exchange of an unactivated C(sp^3^)–H bond; (**b**) two examples of site- and enantioselective incorporation of deuterium into organic compounds.

Photoredox catalysis has also been applied for the in situ deuteration of thiols with D_2_O, which provides an easy and effective way to incorporate deuterium in stable C-D bonds, a process that, as already mentioned in the previous section, allows the deuterium enrichment for the metabolic stabilization of drugs [105]. This subject has been recently reviewed [106], and here we mention a couple of recent examples. The scope of the radical H/D exchange of unactivated C(sp^3^)–H bonds as well as the multi-deuteration of C(sp^3^)–H bonds with D2O as a cheap deuterium source was studied by utilizing a synergistic photoredox catalysis and organocatalysis system in detail, including a scale-up experiment [107]. Figure 41a shows the optimized example of a remote site-selective radical C(sp^3^)–H deuteration of PMP amides by utilizing a photoredox catalytic system made on an iridium(III) complex with Bu_4_N^+^ (BuO)_2_P(O)^−^ together with an RSSR/D_2_O system [107]. A visible-light-mediated catalytic asymmetric radical deuteration at non-benzylic positions was also developed through a series of radical addition reactions of mainly *N*-heterocyclic carbene-borane complexes, and of silicon and phosphine radicals, to exocyclic double bonds coupled with asymmetric deuteration of the resulting radical with a chiral thiol/D_2_O system [108]. Two examples are shown in Figure 41b, the first is the optimized conditions for the addition of *N*-heterocyclic carbene–borane complex onto exocyclic olefin, and the second is a deuterosilylation with (TMS)_3_SiH. In both cases, cysteine-derived β-turn-containing peptide (R*SH) as the deuterium atom transfer catalyst and readily available 4DPAIPN as the organophotocatalyst in a binary solvent mixture of toluene and D_2_O (3:1) were used (see the right side of Figure 41b for the structures). The mechanism of the reaction was studied theoretically, and a favored transition state was proposed for the origin of enantioselectivity, while the study of the scope of the reaction indicated greater than 95:5 enantiomeric ratios in many cases [108].

**Figure 42:**
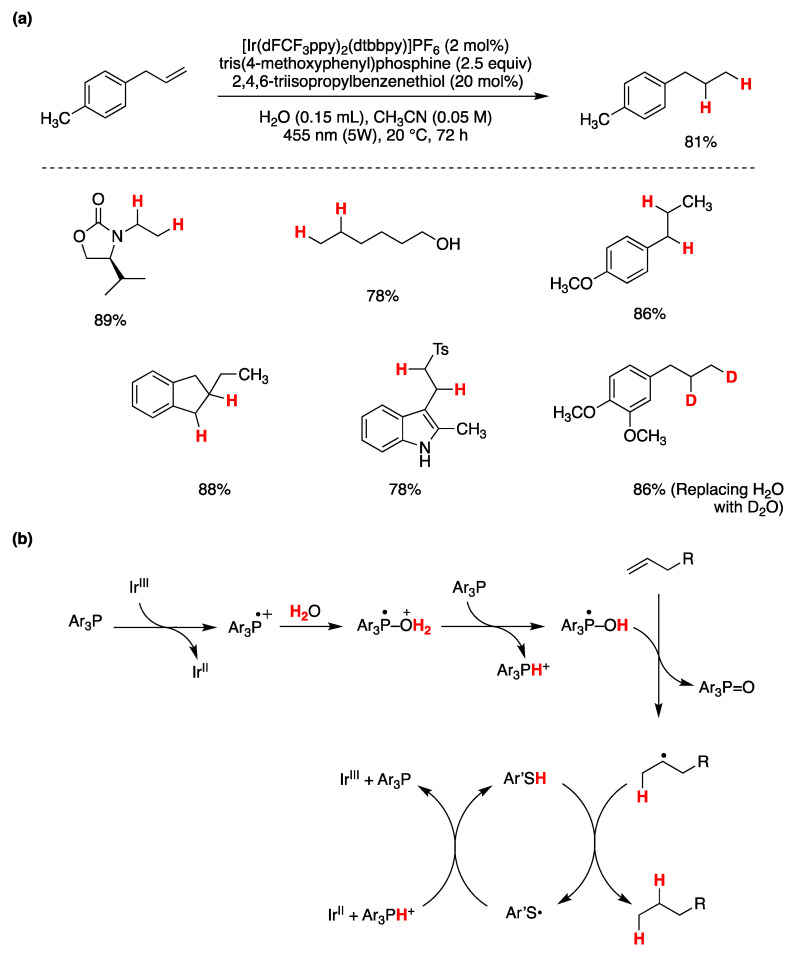
(**a**) Optimized conditions for the photocatalytic phosphine-mediated water activation for radical hydrogenation and selected examples; (**b**) proposed mechanistic scheme.

It is also worth mentioning the most recent report on phosphorous chemistry by the group of A. Studer [109] described an interesting photocatalytic system based on a phosphine-mediated water activation for radical hydrogenation. Figure 42a shows the optimized conditions for this transformation and some selected examples of alkene hydrogenation. It is based on an iridium(II) photocatalyst that generates the radical Ar_3_P(^•^)–OH as an intermediate, which can be considered a formal ‘free’ H atoms donor. Arylthiols are used as catalytic co-reductants for the radical hydrogenation of π systems. This co-catalysis approach ensures that both H atoms of water can be used as H-atom donors in the reduction of π systems, as shown in Figure 42b. Indeed, control experiments revealed that H_2_O is the exclusive hydrogen source in the hydrogenation and the solvent (acetonitrile) is not involved. The preparation of a few deuterated compounds using cheap D_2_O was also reported (see Figure 42a for an example) [109].

## 7. Bioinspiration from Biological Reactivity

There are many examples in the literature of bioinspired reactions in organic synthesis and some of them are connected with free radical reactivity [34,161]. Knowledge of free radical reactivity taking place in biological processes can be very important for organic chemistry in two main directions: (i) the design of biomimetic models to simulate free radical damage and provide molecular libraries for mechanistic and biomarker discovery, and (ii) the inspiration for new synthetic methods based on the same mechanisms that nature uses to prepare biomolecules. In this section, we report two examples of such approaches of interest in our laboratory: the synthesis of purine 5′,8-cyclo-2′-deoxynucleosides via radical cyclization and the reduction of carbonyl moieties by disulfide radical anion.

### 7.1. Purine 5′,8-Cyclo-2′-deoxynucleosides

**Figure 43:**
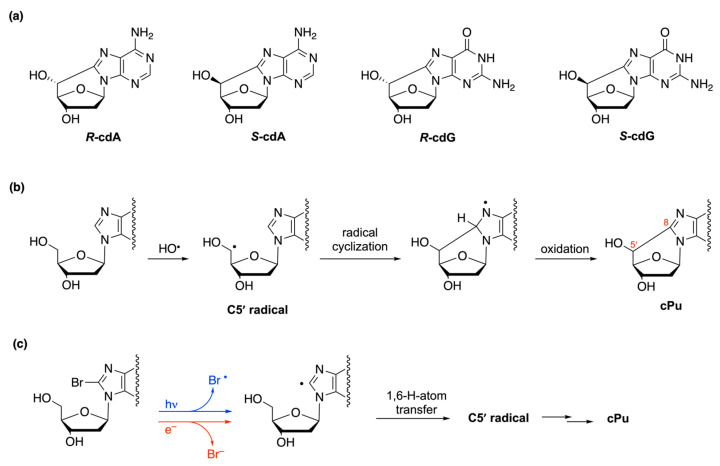
(**a**) Structures of 5′,8-cyclo-2′-deoxyadenosine (cdA) and 5′,8-cyclo-2′-deoxyguanosine (cdG) in their 5′*R* and 5′*S* diastereomeric forms. (**b**) Bioinspired radical transformations for the synthesis of 5′,8-cyclopurines (cPu). (**c**) Radical cascade reaction that mimics the DNA damage for the synthesis of 5′,8-cyclopurines (cPu).

Purine 5′,8-cyclo-2′-deoxynucleosides or 5′,8-cyclopurines (cPu) are important modified nucleosides that represent tandem-type lesions observed among the DNA modifications and have been identified in mammalian cellular DNA in vivo [162,163,164,165]. Figure 43a shows the four cPu structures, i.e., 5′,8-cyclo-2′-deoxyadenosine (cdA) and 5′,8-cyclo-2′-deoxyguanosine (cdG) in their 5′*R* and 5′*S* diastereomeric forms, whereas Figure 43b shows the formation of the C5′ radical by H-atom abstraction from HO^•^ radical and the subsequent steps of the mechanism of cPu formation.

Biomimetic experiments for the cPu formation and related mechanism were performed using γ-radiolysis as HO^•^ radical-generating sources. γ-Radiolysis of neutral water leads to the following primary reactive species: solvated electrons (e^−^_aq_), hydroxyl radicals (HO^•^), and hydrogen atoms (H^•^). In an N_2_O-saturated solution (~0.02 M of N_2_O), e^−^_aq_ are efficiently transformed into HO^•^ radicals, and therefore HO^•^ and H^•^ account for 90% and 10%, respectively, of the reactive species. Under these conditions, it was found that both dG and dA afford ~10% yields of cdG or cdA in *R*/*S* product ratios of 8.3:1 and 6:1, respectively [166,167,168]. The reactivities of HO^•^ with dG or dA are unselective and the obtained yields correspond to the H-atom abstraction from C5′–H which is ~10% [25]. Hydrated 5′-aldehydes were formed instead of the cPu in the presence of oxygen, indicating the trapping of C5′ radicals by oxygen prior to cyclization [169]. The rate constants of radical cyclization were determined by time-resolved spectroscopy to be 1.6 × 10^5^ and 6.9 × 10^5^ s^−1^ at room temperature for dA and dG, respectively (Figure 43b) [168,170].

Synthetic procedures for cPu nucleosides were established starting from the corresponding 8-bromopurine derivatives in aqueous solutions, with a radical cascade mechanism that mimics DNA damage (Figure 43c). Indeed, UV photolysis of 8-Br-2′-dG afforded cdG in 26% yield and an *R*/*S* ratio of 8:1 [166], whereas the gamma-radiolysis of 8-Br-2′-dA in the presence of K_4_Fe(CN)_6_ afforded of cdA in 67% yield (based on the starting material conversion) and an *R*/*S* ratio of 6:1 [168]. It is worth mentioning that the *pro*-5′*R* conformer is stabilized in water when the 3′OH and 5′OH groups are free, whereas the *pro*-5′*S* conformer prevails in aprotic solvents when the 3′OH and 5′OH groups are protected with a TBDMS group [171,172].

Analogous reactions have been employed: (i) for the isotopic synthesis of ^15^*N*_5_-labeled cPu derivatives necessary as reference material for quantification of oxidative DNA lesions in biological samples by liquid chromatography-tandem mass spectrometry [173,174,175]. Such quantification has been a subject of interest in our laboratory for the last 5 years; we provided evidence for cPu formation in cells [176,177,178,179], animals [180,181], and humans [182] as valuable biomarkers for free radical activity. (ii) for the synthesis of the phosphoramidite synthones of the cPu [183] for further incorporation to model DNA for biological studies [184,185,186,187].

### 7.2. Disulfide Radical Anion as a Reductant

**Figure 44:**
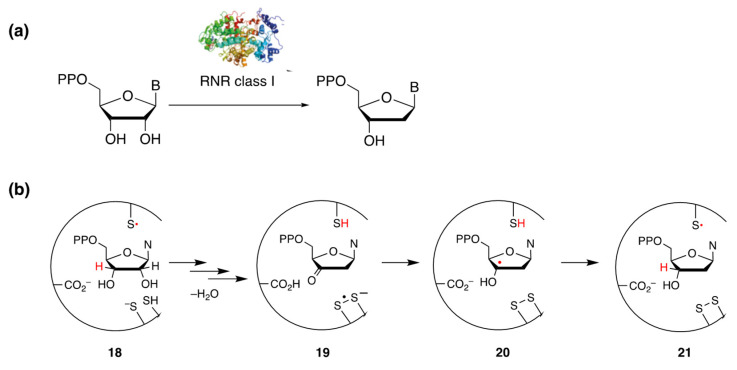
(**a**) Transformation of ribonucleotides to 2′-deoxy-ribonucleotides by ribonucleotide reductases class Ia, active in eukaryotes and microorganisms (taken from Ref. [190]). (**b**) Some details of the complex radical mechanism at the active site of enzyme; in particular, the reaction **19** → **20** is the reduction of ketone by disulfide radical anion (taken from Ref. [195]).

2′-Deoxy-ribonucleotides diphosphates are the building blocks for the de novo synthesis of DNA. Ribonucleotide diphosphates undergo a selective but complex radical mechanism by the ribonucleotide reductase class I, active in eukaryotes and microorganisms. (Figure 44a) [188]. Figure 44b summarizes this multistep mechanism [188], which initially involves a reversible H-atom abstraction from C3′–H by the thiyl radical generated from one of the cysteine residues at the active site (**18**). The subsequent steps involve the elimination of H_2_O with translocation of the radical center to C-2′, quenching of the newly formed C-2′ radical by another cysteine residue, and formation of a disulfide radical anion (**19**). The reduction of ketone is followed by disulfide radical anion (**19** → **20**) and the restoration of the initial cysteine residue (**20** → **21**). The restoration of the other two cysteine residues occurs by the intervention of thioredoxin reductase (TRR) and NADPH [189].

**Figure 45:**
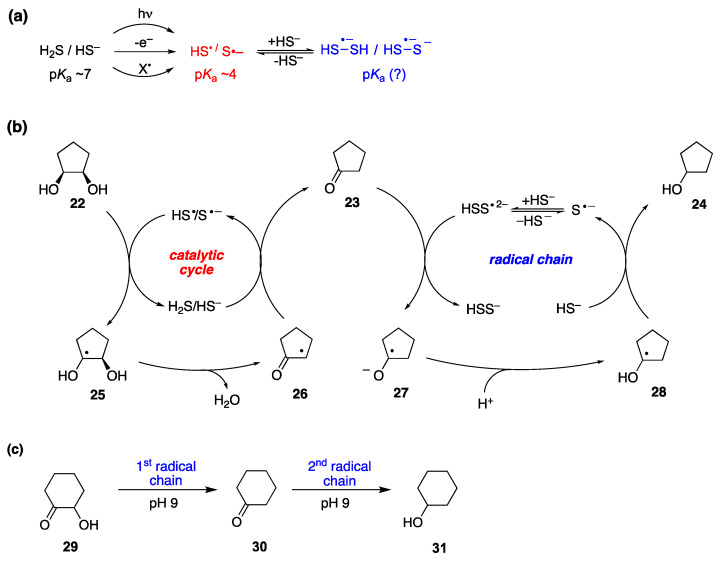
(**a**) Hydrogen sulfide (H_2_S/HS^−^) affords the sulfhydryl radical (HS^•^/S^•−^) by a variety of methods and adds reversibly to the parent compound to form the dimeric radical anion. (**b**) Dual catalytic/radical chain mechanism for the deoxygenation of cis-1,2-cyclopentanediol (**22**) to cyclopentanol 24) via cyclopentanone (**23**) in aqueous solutions (taken from Ref. [190]). (**c**) Dual radical chain mechanism for the deoxygenation of 2-hydroxycyclohexanone (**29**).

In chemical synthesis, the selective deoxygenation of a 1,2-diol to alcohol is a difficult conversion that can be accomplished by dehydration of a 1,2-diol to an aldehyde or ketone followed by a reduction. For example, the Barton-McCombie approach is an example of multistep reactivity that is applicable to sugar substrates and involves protection and derivatization to an intermediate that undergoes a radical-mediated deoxygenation reaction [36,190]. The enzymatic transformation shown in Figure 44 has been taken as inspiration for using sulfur radical chemistry in the cis-1,2-cyclopentanediol (**18**) transformation to cyclopentanol (**20**) as well as the reduction of carbonyl compounds by disulfide radial anion [191] (Figure 45b).

The method employs H_2_S/HS^−^ generated in situ by dissolving Na_2_S in water at suitable pH and the corresponding transient species HS^•^/S^•−^ and HSSH^•−^/HSS^•2−^ (Figure 45a) [192]. The initial HS^•^/S^•−^ radical abstracts an H atom from cis-1,2-cyclopentanediol (**22**) to give the radical **25**, which undergoes a β-C—O scission and H_2_O elimination to give radical **26** that completes the catalytic cycle by reacting with H_2_S/HS^−^, regenerating HS^•^/S^•−^ and forming cyclopentanone (**23**) (left side of Figure 45b). The ketone **23** is reduced by the dimeric radical anion HSSH^•−^/HSS^•2−^ to the radical anion **6**, followed by protonation and H-atom abstraction of **28** from H_2_S/HS^−^ affords the product cyclopentanol (**24**) completing the radical chain (right side of Figure 45b). pH Dependence experiments demonstrated that the catalytic cycle is more efficient under acidic conditions (pH < 7) whereas the radical chain is more effective under basic conditions (pH > 7) [191]. Indeed, a variety of carbonyl compounds are reduced to the corresponding alcohols in excellent yields (90–99%) using UV light for 1h at pH 9 and 42 °C [191]. It is also worth mentioning that the reduction of 2-hydroxycyclohexanone (**8**) under the same conditions proceeds via a dual radical chain mechanism, affording first the ketone **9** and then the alcohol **10** (Figure 45c). The reduction potential *E*(HSS^−^/HSS^•2−^) is estimated to be −1.13 V [193], indicating the efficiency of the species HSS^•2−^ for one-electron transfer to a carbonyl group. The reactive species derived from H_2_S and the reported mechanistic steps in Figure 45 can also be discussed in terms of prebiotic photochemical transformations, with H_2_S and UV light being both present in the primitive earth environment [194,195].

**Figure 46:**
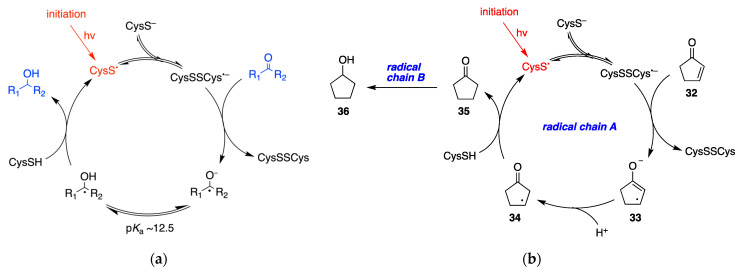
(**a**) The radical chain reaction for the reduction of ketones to the corresponding alcohols; (**b**) the mechanism for the reduction of 2-cyclopenten-1-one (**19**) involves dual radical chain reactions A and B (taken from Ref. [195]).

The bioinspired conversion of ketones to alcohols was also achieved by the intermediacy of disulfide radical anion of cysteine (CysSSCys)^•−^ in water [196]. A variety of ketones are reduced to the corresponding alcohols in excellent yields (91–99%) using UV light (low-pressure Hg lamp, 5.5 W), for 1h, at pH 10.6 and 42 °C, in N_2_-saturated aqueous solutions. However, a high concentration of cysteine and pH 10.6 are necessary for shifting the equilibrium to (CysSSCys)^•−^ and providing high-yielding reactions. The reaction mechanism for the reduction of a ketone to the corresponding alcohol is also shown in Figure 46a. The ketone is reduced to ketyl radical anion via single-electron transfer, followed by protonation from the aqueous medium and H-atom abstraction from CysSH affording the alcohol, thus completing the radical chain mechanism. Figure 46b shows the reduction of 2-cyclopenten-1-one (**32**) under identical experimental conditions. The reported time profile of the reduction of compound **32** at pH 10.6 shows clearly that the reaction **32** → **36** occurs stepwise, with the formation of the cyclopentanone (**35**) as the intermediate stable product, i.e., proceeds via a dual radical chain mechanism affording first the ketone **35** and then the alcohol **36**.

The standard reduction potential *E*^0^(CysSSCys/CysSSCys^•−^) = −1.38 V [197,198] indicates the efficiency of CysSSCys^•−^ for single-electron transfer to a carbonyl group. Recently, the standard reduction potential of a variety of alkyl and aromatic disulfides has been reported [199]. The absorption spectra of (CysSSCys)^•−^ have a λmax at 420 nm [200]; pulse radiolysis studies of the decay of (CysSSCys)^•−^ at various concentrations of ketones, determined the rate constants of three cyclic ketones to be in the range of 104–105 M^−1^ s^−1^ at ~22 °C [195].

## 8. Conclusions

An overview of radical reactions in water or aqueous media achieved in the last two decades has been presented. The number of applications of radical reactions “in water” and “on water” has been continuously increasing, either using known radical chemistry or bioinspired processes from nature, towards biomarker development. Many methodologies of coordination-induced water H–OH bond weakening and photocatalysis using water as the solvent or reagent have been developed, finding new radical chain processes. Some of them have already been applied in the development of novel synthetic methodologies. In addition, the emergence of molybdenum, germanium, bismuth, and phosphorous inducing the lowering of H–OH or H–OR BDEs enlarges this promising field, and is expected to provide other useful and novel synthetic applications in the near future. It is worth pointing out that chemical reactivity in nature occurs essentially in water and there is certainly much more to learn from natural chemical processes proceeding through single-electron transfer in this medium. It is very important to establish biomimetic models, which allow the experiments to be performed in a simplified environment, but suitably designed to be in strict connection with cellular conditions in terms of complexity and kinetics. The biomimetic approach is a convenient tool with clear outputs, such as (i) a better comprehension of the biological pathways in health and diseases; and (ii) the development of molecular libraries that can be used as mechanistic proofs of new chemical processes as well as biomarkers of metabolic pathways.

## Figures and Tables

**Table 1:** Rate constants for the reaction of α-hydroxyalkyl radicals with 2-mercaptoethanol in water as the solvent ^1^.

Alkyl Radical	*k*_H_, M^−1^ s^−1^
HOCH_2_^•^	1.3 × 10^8^
HOC(^•^)Me	2.3 × 10^8^
HOC(^•^)Me_2_	5.1 × 10^8^

^1^ At room temperature.

## Data Availability

No new data were created.
